# Structural Characterization of *Arabidopsis thaliana* NAP1-Related Protein 2 (AtNRP2) and Comparison with Its Homolog AtNRP1

**DOI:** 10.3390/molecules24122258

**Published:** 2019-06-17

**Authors:** Ashish Kumar, Ajit Kumar Singh, Ruchir Chandrakant Bobde, Dileep Vasudevan

**Affiliations:** 1 Institute of Life Sciences, Bhubaneswar 751023, Odisha, India; mail2ashish.bt@gmail.com (A.K.); singh.ajit325@gmail.com (A.K.S.); rckngb30593@gmail.com (R.C.B.); 2 Manipal Academy of Higher Education, Manipal 576104, Karnataka, India; 3 Regional Centre for Biotechnology, Faridabad 121001, Haryana, India

**Keywords:** *Arabidopsis thaliana*, histone chaperone, nucleosome assembly protein, NAP, NAP1-related protein 2, NRP, NRP1, NRP2, crystal structure, SAXS

## Abstract

Nucleosome Assembly Protein (NAP) is a highly conserved family of histone chaperones present in yeast, animals, and plants. Unlike other organisms, plants possess an additional class of proteins in its NAP family, known as the NAP1-related proteins or NRP. *Arabidopsis thaliana* possesses two NRP isoforms, namely AtNRP1 and AtNRP2, that share 87% sequence identity. Both AtNRP1 and AtNRP2 get expressed in all the plant tissues. Most works in the past, including structural studies, have focused on AtNRP1. We wanted to do a comparative study of the two proteins to find why the plant would have two very similar proteins and whether there is any difference between the two for their structure and function as histone chaperones. Here we report the crystal structure of AtNRP2 and a comparative analysis of its structural architecture with other NAP family proteins. The crystal structure of AtNRP2 shows it to be a homodimer, with its fold similar to that of other structurally characterized NAP family proteins. Although AtNRP1 and AtNRP2 have a similar fold, upon structural superposition, we find an offset in the dimerization helix of the two proteins. We evaluated the stability, oligomerization status, and histone chaperoning properties of the two proteins, for a comparison. The thermal melting experiments suggest that AtNRP2 is more stable than AtNRP1 at higher temperatures. In addition, electrophoretic mobility shift assay and isothermal titration calorimetry experiments suggest histone binding ability of AtNRP2 is higher than that of AtNRP1. Overall, these results provide insights about the specific function and relevance of AtNRP2 in plants through structural and biophysical studies.

## 1. Introduction

The nuclear processes like DNA replication, transcription, DNA repair, and DNA recombination require a highly regulated disassembly and assembly of nucleosomes, which form the basic repeating units of chromatin. In this context, chromatin remodeling is a crucial step, and it is brought about by a variety of histone modifying enzymes, and ATP-dependent chromatin remodeling factors [1] in coordination with a heterogeneous group of ATP-independent chromatin remodelers known as the histone chaperones [2,3,4]. The histone chaperones are a group of acidic proteins that are implicated in preventing non-productive aggregation between highly positively charged histones and negatively charged DNA molecules in the protein-dense environment of the eukaryotic nuclei [4,5,6]. In addition to shielding the charge of histones, histone chaperones are involved in the shuttling of histones inside the nucleus, as well as in nucleosome assembly and disassembly processes. This diverse group of proteins could be classified based on the functions they perform, the histones they interact with, or on their oligomeric status and stoichiometry of histone-binding [7].

Nucleosome Assembly Protein (NAP), is a highly conserved and well-studied family of histone chaperones in yeast [8,9], animals [10,11], and plants [5,12,13,14]. NAP1 is an extensively characterized member of the NAP family of histone chaperones, in terms of its structure, histone-binding activity, and chromatin assembly functions [3]. It binds with all the histones, H2A, H2B, H3, and H4 in vitro, but is explicitly recognized as an H2A-H2B binding histone chaperone [2,3,5,6]. It is encoded by a single gene in case of *Saccharomyces cerevisiae* but belongs to a multigene family in mammals and plants [5]. *Arabidopsis*, as well as rice, contain four homologs of NAP1 proteins, NAP1;1, NAP1;2, NAP1;3 and NAP1;4 [14,15,16] and two distantly related proteins called NAP1-Related Proteins (NRP1 and NRP2) [5,12,17,18].

NRP1 and NRP2 are histone H2A-H2B interacting proteins, closely related to animal SET/TAF-1β and are involved in functions such as histone chaperoning [5,19], protein phosphatase 2A (PP2A) inhibition [20], cell cycle regulation, root meristem formation, heat tolerance, protection of cells from DNA damage during genotoxic stresses, and nucleosome assembly activity in vitro [18,21,22]. The NRPs show a ubiquitous expression, similar to the classical NAP domain-containing proteins NAP1;1, NAP1;2 and Nap1;3 [15,16]. The function of both AtNRP1 and AtNRP2 were initially identified by reverse genetics approach, where *Atnrp1-1* and *Atnrp2-1* double mutants were shown to have their cell cycle arrested at the G2/M phase, resulting in the disordered cellular organization at the root tip, and leading to impaired post-embryonic root growth [5]. Recently, it has been shown that AtNRP1 gets enriched at the *GLABRA2* (GL2) promoter region, which plays a crucial role in root patterning by interacting with the WEREWOLF (WER) transcription factor, leading to local histone eviction and ultimately nucleosome loss at the GL2 promoter resulting in its activation [18]. Thus, in the case of NRP double mutants, the GL2 promoter remains silenced, and the root patterning gets impaired [18]. The current understanding of the stem cell niche (SCN) function during continuous post-embryonic root development has been further improved based on genetic studies done with AtNRP1/2 and CAF1. These studies show that the histone chaperones AtNRP1/2 and CAF1 work cooperatively to regulate proper genome transcription, to maintain genome integrity, and to sustain DNA replication as well, thereby controlling SCN function [23]. Both AtNRP1 and AtNRP2 have also been shown to be involved in maintaining the genome stability during somatic homologous recombination process, and their double mutants were shown to have enhanced DNA damage [5,21]. Another recent report explains the mechanism of AtNRP1 action during the DNA damage response wherein Cytochrome C (Cc) binds competitively to the histone-binding regions of AtNRP1, thereby obstructing its histone chaperoning activity and allowing the repair process to occur. This activity of AtNRP1 is very similar to the role of animal SET/TAF-1β proteins [22]. In addition to their remarkable role in regulating development and genotoxic stress conditions, NRPs are being studied in relation to heat stress conditions as well [20]. Additionally, more recently, these were shown to be essential in regulating several defense-related genes during infections by necrotrophic and biotrophic pathogens [16]. The increased expression of WRKY transcription factors in overexpression lines of AtNRP1 (*nrp1ox*) was attributed to the higher expression levels of pathogenesis-related (PR) genes and thereby, increased tolerance towards pathogenic infections [16].

Alterations in gene expression profiles of *Atnrp1-1* mutant, *Atnrp2-1* mutant, and *Atnrp1-1 Atnrp2-1* double mutant have been studied in the past, showing differential expression of 10 genes in *Atnrp1-1* mutant, 483 genes in *Atnrp2-1* mutant, and 102 genes in *Atnrp1-1 Atnrp2-1* double mutant [5]. AtNRP2 seems to have a broader activity base, as a considerably higher number of genes get differentially expressed in the case of *Atnrp2-1* single mutant. In addition, this suggests that the function of AtNRP2 is only partially redundant to that of AtNRP1, supporting the view that the control of gene expression by NRPs could be dependent on their interactions with other factors [5,16]. More recently, the crystal structure of AtNRP1 was determined, which revealed an ellipsoid homodimer with a “head-phone” fold similar to ScNAP1 protein [18]. AtNRP1 has been shown to interact with individual histones H2B, H3 and H4, H2A-H2B dimers, and H3-H4 tetramers with different binding affinities [22]. However, neither structural nor binding characterizations have been carried out for AtNRP2. With the above background, through this work, we aim to understand the structure-function features of AtNRP2 in comparison to AtNRP1.

Through phylogenetic analysis, we show that AtNRP2 is more abundantly distributed among land plants than AtNRP1. Additionally, NRP2 seems to have evolved independently of NRP1 in *Arabidopsis thaliana*, further supporting its significance in the plant. Here we report the crystal structure of AtNRP2, which reveals a fold similar to that of NAP family proteins, with minor differences as compared to AtNRP1 and SET/TAF-1β. However, we observe some significant differences for both AtNRP1 and AtNRP2 with other NAP family proteins, which probably would explain the differences in the histone-binding capabilities of NAP1 and NRPs. We find that AtNRP2 and AtNRP1 are quite stable at physiologically relevant higher temperatures, suggesting them to be crucial during hyperthermal stress conditions. Finally, we have compared the histone-binding capabilities and found both AtNRP1 and AtNRP2 to interact with histone H2A-H2B dimer in vitro. Interestingly, we find that binding affinity to histone H2A-H2B is significantly higher for AtNRP2 than for AtNRP1. We also show that both AtNRP1 and AtNRP2 are capable of nucleosome assembly activity in vitro and thus act as histone chaperones.

## 2. Results

### 2.1. Sequence-Based Molecular Phylogenetic Studies on Plant NRP1 and NRP2

**Figure 1:**
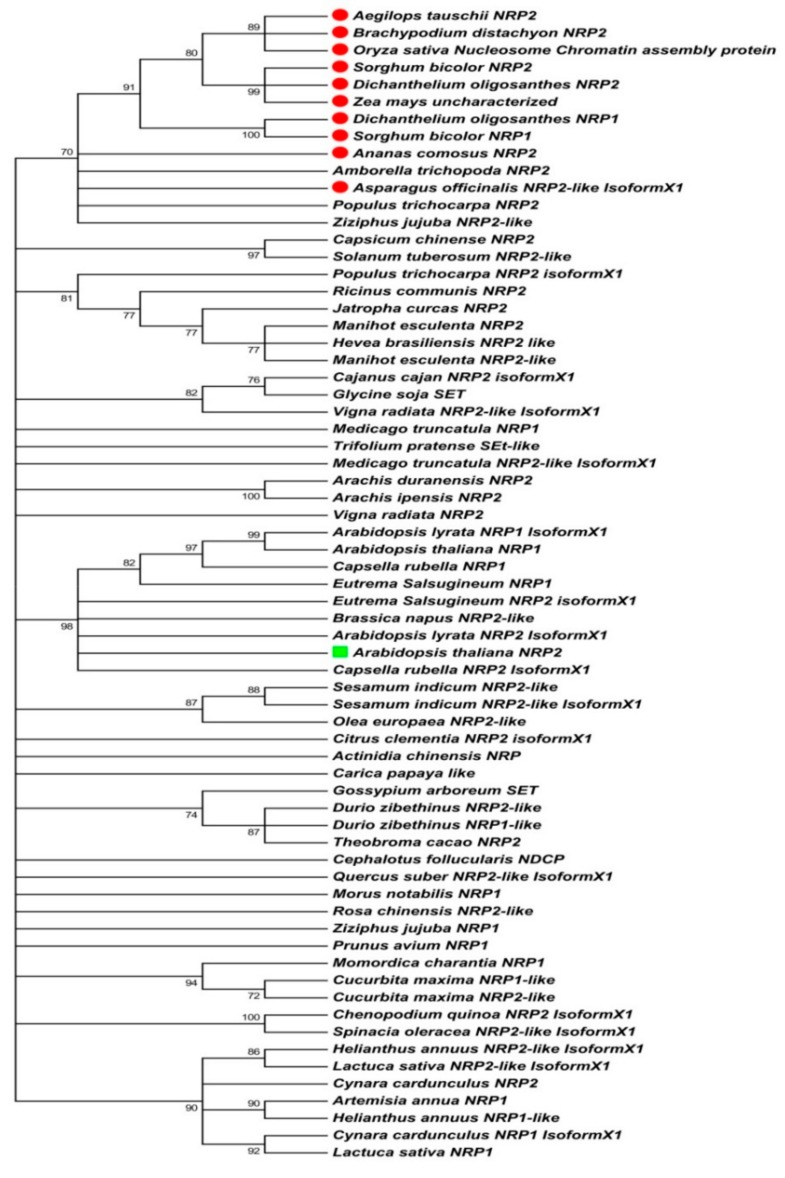
Molecular phylogenetic analysis of NAP1-Related Proteins (NRPs) by maximum likelihood method. The evolutionary history was inferred by using the maximum likelihood method based on the Poisson correction model. The bootstrap consensus tree derived from 1200 replicates was taken to represent the evolutionary history of the taxa analyzed. Branches corresponding to partitions reproduced in less than 70% bootstrap replicates were collapsed. The percentage of replicate trees in which the associated taxa clustered together in the bootstrap test (1200 replicates) are shown next to the branches. Initial tree(s) for the heuristic search were obtained automatically by applying Neighbor-Join and BioNJ algorithms to a matrix of pairwise distances estimated using Jones-Taylor-Thornton (JTT) model, and the topology selected with superior log-likelihood value. The analysis involved 67 amino acid sequences. All positions containing gaps and missing data were eliminated. There were a total of 178 positions in the final dataset. Evolutionary analyses were conducted using MEGA 7.0 software. In the phylogenetic tree, NRPs from monocots are highlighted using a red circle and AtNRP2 with a green square.

Although both NRP1 and NRP2 have similar histone chaperone functions, NRP2 does not show a complete redundant role [5]. This clues it to be important in controlling the expression of various genes in plants, especially during biotic and abiotic stresses. The above fact led us to hypothesize that NRP2 could be functionally more relevant in plants than NRP1. To understand its significance in a plant system, we tried to understand its evolutionary relationship with NRP1 within all the land plants. For this purpose, we selected AtNRP2 (NP_564063.1) and OsNRP2 (XP_015627496.1) protein sequences as a seed sequence to find similar proteins in both dicots and monocots, respectively. The identical proteins from the various dicots and monocots were selected by BLAST search against the non-redundant protein sequence database for all land plants (Taxid: 3193). For multiple sequence alignment and phylogenetic analysis, the NRP2 sequences from those species were selected, which were at least 70% identical. The species containing both NRP1 and NRP2 were selected based on the criteria where NRP2 should be with 70% or more sequence identity. Multiple sequence alignment was carried out by the Multiple Sequence Comparison by Log- Expectation (MUSCLE) program [24], from the software suite Molecular Evolutionary Genetics Analysis version 7.0 (MEGA 7.0) (Pennsylvania State University, University Park, PA, USA) and further, a phylogenetic tree was constructed using MEGA 7.0 [25,26] by using the maximum likelihood method [27]. The sequence analysis shows that NRP2 sequence ranges from 190 amino acids (*Dichanthelium oligosanthes*) to 275 amino acids (*Amborella trichopoda*). A total of 67 NRP sequences (NRP1 and NRP2) from various plant species were analyzed. NRP2 was found to be more frequent in occurrence among the plants compared to NRP1 (Supplementary Table S1). Several plants had only NRP2. There were only few plants containing both NRP1 and NRP2 (*Arabidopsis lyrata, Arabidopsis thaliana, Capsella rubella, Cucurbita maxima, Cucurbita moschata, Cynara cardunculus, Dichanthelium oligosanthes, Durio zebethinus, Eutrema salsugineum, Helianthus annuus, Lactuca sativa, Manihot esculenta, Medicago truncatula, Sorghum bicolor* and *Ziziphus jujuba*) and very few containing only NRP1 (*Artemisia annua, Momordica charantia, Morus notabilis, Prunus avium, Prunus persica,* and *Prunus yedoensis*), which again supports the previous finding [5,16] that NRP2 is functionally more important than NRP1 in plants. In addition, out of the selected 56 species, we observed that NRP2 is extensively distributed among dicots, and only 10 monocots were found closer to the selected NRP2 sequences from the various dicots (Figure 1). All the monocots form a distinct clade from that of dicot plants, except for one dicot species, i.e., *Amborella trichopoda*. According to APG III and the current APG IV flowering plant classifications, *A. trichopoda* belongs to Amborellaceae family with a single genus *Amborella,* and it is considered as a very basal and extant flowering plant, nearer to monocots in origin [28]. Due to its primitive nature, *A. trichopoda* NRP2 gets grouped along with the monocot clade in our phylogenetic classification, which shows its similarity and relatedness to monocots. The NRP-containing monocots are mostly from the two orders—Poales and Asparagales; however, in dicots, it includes orders from different clades like Asterids, Rosids, and Malvids. The phylogenetic tree also shows NRP2 in a separate group than NRP1, which indicates that NRP2 has evolved independent of NRP1, most probably by gene duplication events. The nearest evolutionary homologs of AtNRP2 are *Brassica napus* NRP2 and *Capsella rubella* NRP2 which are 87.9% and 84.7% identical, respectively to AtNRP2. In case of some plants, the nomenclature of the proteins is ambiguous as these are called SET proteins (*Glycine soja, Gossypium arboreum*, and *Trifolium pratense*)or NAP domain-containing protein (*Cephalotus follicularis*) or Nucleosome chromatin assembly protein (*Oryza sativa indica*). Since these proteins are more homologous to AtNRP2, they could be considered as NRP2 in these species.

In addition to the NRP sequences examined by the phylogenetic analysis, we also investigated the phylome of NAP domain containing proteins using the AtNRP2 sequence (NP_564063.1) as the seed sequence and using PhylomeDB v4 database (http://phylomedb.org) [29]. The phylogenetic tree obtained from the phylome database (Supplementary Figure S1) shows the evolution of various NAP domain-containing proteins from multiple organisms including algae, plants, and animals. It shows that the NAP and NAP related proteins (NRPs and SET) have evolved separately because of gene duplication in a single ancestral gene followed by independent speciation events. Here also, we see AtNRP1 and AtNRP2 have evolved independently of each other because of gene duplication and speciation events. This finding is in agreement with our phylogenetic analysis done using the maximum likelihood method. It also shows that AtNRP1 and AtNRP2 are homologous proteins and further lead to the identification of evolutionarily closer orthologs like those from *Mimulus guttatus* and *Vitis vinifera* which we did not observe in our phylogenetic analysis. We have found that *Glycine max* NRP2 has the smallest NAP domain among all angiosperms. However, *Physcomitrella patens*, which is a bryophyte has the smallest NAP domain-containing protein and the structural and functional studies on these could give us a better understanding of the evolution of NAP domain-containing proteins across the plant kingdom. Based on all these findings, we further hypothesize that in addition to the evolutionary differences, NRP2 might show some structural and functional differences with NRP1 and other NAP domain-containing proteins.

### 2.2. Expression, Purification, and Estimation of the Oligomeric Status of AtNRPs

**Figure 2:**
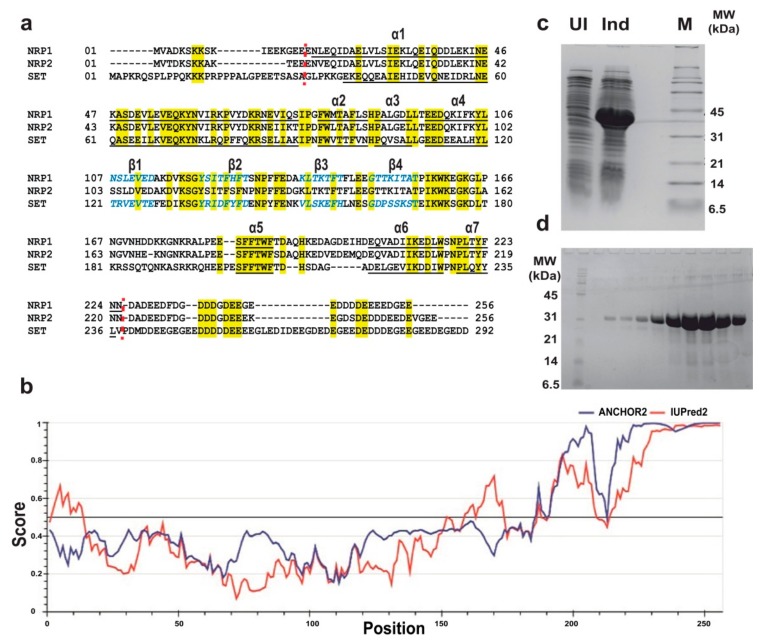
Expression, purification, and sequence analysis for AtNRP2. (**a**) Sequence alignment of AtNRP2 with AtNRP1 and HsSET/TAF-1β. All the conserved residues are shown in yellow. Secondary structures of known structures are shown; wherein, α-helices are underlined, and β-sheets are in blue and italics. Red dotted lines mark the regions where AtNRP2 was truncated based on the secondary structure information of the other two proteins. (**b**) IUPRED prediction of disordered regions of AtNRP2. (**c**) SDS-PAGE imageshowing the expression of AtNRP2 NT/CT upon IPTG induction. The lanes are marked as UI = uninduced, Ind = Induced, and M = Marker. (**d**) SDS-PAGE image showing the purified peak fractions of AtNRP2 NT/CT from gel-filtration chromatography using HiLoad 16/600 Superdex 200pg column (GE Healthcare).The extreme left lane has the marker, and the remaining lanes have the fractions of the protein from gel-filtration.

**Figure 3:**
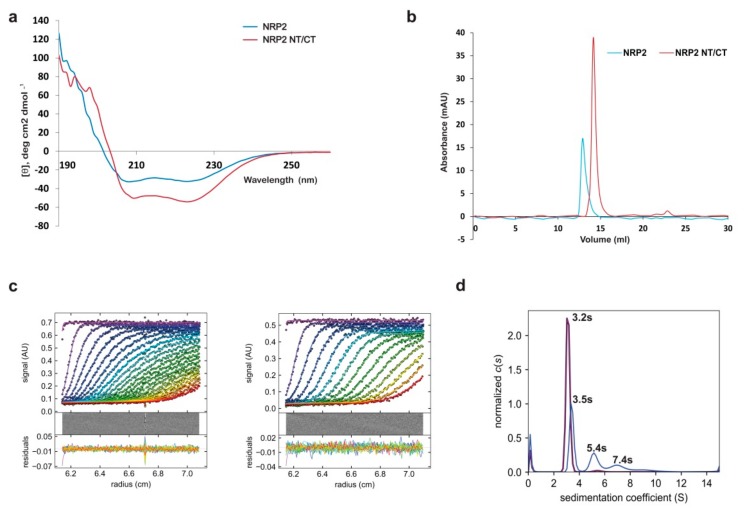
Secondary structure and oligomerization status determination of AtNRP2. (**a**) The near-UV Circular dichroism (CD) spectroscopy profile of full-length AtNRP2 and AtNRP2 NT/CT, showing them to be predominantly α-helical in composition. (**b**) Analytical gel-filtration chromatogram of full-length AtNRP2 and AtNRP2 NT/CT. The elution volumes obtained were nearly 12.9 and 14.0 mL, respectively, which correspond to the sizes bigger than those of the dimers. (**c**) Sedimentation velocity analytical ultra-centrifugation of AtNRP2 (left panel) and AtNRP2 NT/CT (middle panel). (**d**) The plot shows the fitting on c(s) analysis and the distribution of sedimentation coefficients for both the proteins respectively (right panel). Peaks in blue represent full-length AtNRP2 and peaks in purple represent AtNRP2 NT/CT. The higher ‘s’ value corresponds to bigger molecules.

Determining the construct boundaries to enhance the success rate of protein crystallizability is a common practice [30,31,32,33,34,35]. To help the crystallization of AtNRP2, we analyzed the propensity of disordered regions in full-length NRP2 sequences using IUPRED and ANCHOR2 predictors [36,37,38,39]. The original full-length AtNRP2 and AtNRP1 constructs were in a pET22b(+) vector with a non-cleavable C-terminal His-tag. The initial few residues at the N-terminus and more than 30 residues at the C-terminus were predicted to be intrinsically disordered in the full-length AtNRP2 (Figure 2b). Based on this prediction and further alignment with the sequences of AtNRP1 and HsSET/TAF-1β for which crystal structures are known (Figure 2a), we decided to truncate the first 13 residues from the N-terminus and 35 residues from the C-terminus of AtNRP2. Towards this end, the open reading frame coding for the truncated version AtNRP2_14-223_ was cloned into a pGEX-6P-1 vector for expression with a cleavable N-terminal Glutathione S-transferase (GST) -tag. Similarly, the AtNRP1_14-226_ truncated version was also prepared for expression with N-terminal GST-tag. The proteins AtNRP1-His, AtNRP2-His, GST-AtNRP1_14-226_, and GST-AtNRP2_14-223_ (Figure 2c) were all expressed in *Escherichia coli* BL21 (DE3) cells by Isopropyl β-d-1-thiogalactopyranoside (IPTG) induction. The full-length proteins with His-tag were purified by His-trap affinity purification, followed by gel-filtration chromatography. The truncated GST-fusion proteins were purified by GST-affinity purification, followed by on-column cleavage of GST-tag and further gel-filtration chromatography (Figure 2d). The truncated proteins are hereafter denoted as AtNRP1 NT/CT and AtNRP2 NT/CT, to indicate that both the N and C –termini of the proteins are truncated. Both full-length AtNRP2 and AtNRP2 NT/CT had the typical α-helical conformation, as confirmed using circular dichroism (CD) spectroscopy (Figure 3a). AtNRP1 and AtNRP1 NT/CT also showed the typical α-helical conformation (Supplementary Figure S2). Thus, we confirmed that the truncation of N-and C-termini did not result in any alteration of the overall secondary structure of the protein.

AtNRP1, which is 87% identical with AtNRP2, has recently been reported to exist as a homodimer in its crystal structure [18].The other similar proteins like SET/TAF-1β and ScNAP1 also predominantly exist as homodimers in solution [9,40]. Based on the similarity in size and sequence of AtNRP2 with these proteins, we assumed AtNRP2 also to exist as a dimer. However, the preparatory grade gel-filtration profile of AtNRP2 and AtNRP2 NT/CT showed the proteins to elute much earlier than what is expected for a dimer in solution (data not shown). To further investigate their oligomeric status, we performed analytical gel-filtration chromatography (Figure 3b) and calculated the molecular masses using K_av_ values of protein calibration markers. Here again, the proteins eluted earlier than what is expected of a dimer. The calculated molecular masses from analytical gel-filtration chromatography were around 288 and 110 kDa for AtNRP2 and AtNRP2 NT/CT, respectively, which are way higher than the expected size of the respective dimers.

We also performed sedimentation velocity analytical ultra-centrifugation experiment to determine the oligomeric state of the proteins. The c(s) distribution model shows full-length AtNRP2 to be predominantly existing as a dimer in solution with three discrete species at 3.5s, 5.4s, and 7.4s (Figure 3c). When we modeled these species of full-length AtNRP2 using the non-interacting species model, the peak at 3.5s was corresponding to the size of a dimer, and the one at 5.4s to the size of a tetramer. However, we could not model the third species at 7.4s, which could be a higher oligomeric form of the same protein. We performed similar c(s) analysis for AtNRP2 NT/CT also, and it gave only a single discrete species at 3.2s, corresponding to the dimer size of the protein. From c(s) analysis, it was clear that the full-length AtNRP2 could exist in multiple oligomeric conformations. However, AtNRP2 NT/CT was found to exist predominantly as a dimer. In addition, the analytical ultra-centrifugation profile showed AtNRP2 NT/CT to be very homogenous in comparison to the full-length protein (Figure 3c), suggesting it to be a better candidate for crystallization experiments. The frictional ratio determined for both AtNRP2 and AtNRP2 NT/CT was nearly equal to 1.5, showing the proteins to have an elongated shape. The elongated or rod-shaped molecules show increased Stoke’s radius and elute earlier than their predicted elution volume in gel-filtration chromatography. Thus, the early elution of the proteins in gel-filtration chromatography could be attributed to the shape of the molecules. As such, AtNRP2 could be an elongated molecule, similar to other NAP family proteins like ScNAP1 [9], SET/TAF-1β [40], and AtNRP1 [18].

### 2.3. Crystallization of AtNRP2

The sedimentation velocity analytical ultra-centrifugation experiments showed AtNRP2 NT/CT to be more homogeneous than full-length AtNRP2 (Figure 3d) and as such, crystallization experiments were carried out only with AtNRP2 NT/CT. The protein purified to homogeneity (Figure 2d) was concentrated to 20 mg/mL concentration and used for crystallization screening. Although AtNRP2 NT/CT appeared almost 95% homogenous in the ultra-centrifugation experiment, we could not obtain crystals even after extensive screening. Most of the conditions were either precipitating or showed only phase separation.

Subjecting to high temperature has been employed as a method to purify thermostable proteins and to make them homogeneous [41,42]. Moreover, heating of protein samples has been described as a polishing step to improve the diffraction quality of protein crystals [43]. Improperly folded proteins are prone to aggregation and hence are less stable than adequately folded proteins. Heating might help in removing the less stable and misfolded proteins, thereby increasing the homogeneity of the protein sample. A similar approach has also been described to remove heterogeneous fractions from the highly amyloidogenic protein fractions of transthyretin triple mutant wherein an extended incubation at 55 °C increased the homogeneity of proteins and allowed the formation of reproducible and well-diffracting crystals [43]. Results of our thermostability experiments with AtNRP2 (described later) showed it to be a thermostable protein. Thus, we also applied a pre-heating step before protein crystallization. AtNRP2 NT/CT was heated at 40 °C for 10 min and centrifuged at 13,000× *g* for 20 min at room temperature before crystallization screening.

**Figure 4:**
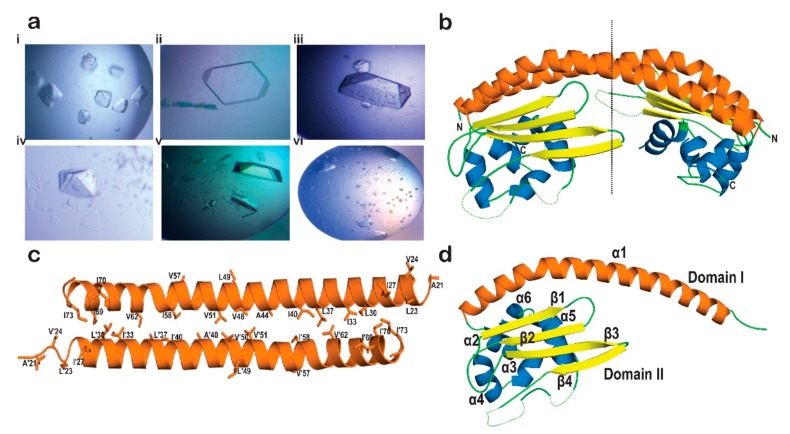
Crystals and structural features of AtNRP2. (**a**) Crystals obtained for AtNRP2 NT/CT in AmSO4 Suite (Qiagen) and tested for diffraction (Conditions (i) 3, (ii) 25, (iii) 27, (iv) 32, (v) 40, (vi) 91). All the crystals were tested for diffraction limits. The best diffraction data was obtained for the big crystal from condition 27, and the collected data was used for structure work. (**b**) The crystal structure of AtNRP2, showing the pseudo-symmetric two-fold axis. (**c**) The distribution of hydrophobic residues along the long dimerization helix of AtNRP2. (**d**) The structure of AtNRP2 monomer, showing the domain organization, where domain I consist of a long dimerization helix (α1) and domain II includes both α-helices and β-strands.

The rapid pre-heat treatment of protein samples helped us to obtain crystals (Figure 4a) in conditions from AmSO4 suite (Qiagen). All the crystals were tested for diffraction quality at the European Synchrotron Radiation Facility (ESRF) beamline ID30A3. The conditions of crystal growth, their cryo-conditions, and diffraction limits are listed in Supplementary Table S2. Most of the crystals diffracted to low resolution. However, we were able to solve the structure of AtNRP2 NT/CT to a resolution of 3.42 Å in the space group H32 by molecular replacement (MR) method. The structure coordinates of AtNRP1 (PDB id: 5DAY) were used as the search model for MR. The data quality and generated maps were good, and we were able to build and refine the structure, thereby achieving reasonable values of R_work_ (27%) and R_free_ (32%), in spite of the data being of low resolution. Data collection and refinement statistics can be found in Table 1.

### 2.4. Crystal Structure of AtNRP2

The overall structure of AtNRP2 revealed an ellipsoid homodimer with a pseudo-two-fold axis, running perpendicular to the long dimerization helix, giving it an earmuff shape (Figure 4b). The overall interface area and the buried surface area of the dimer were estimated as 1466.3 and 2930 Å^2^, respectively. Out of a total of 210 residues in each chain, only 168 residues in chain A and 164 residues in chain B could be built into the structure. Residues in some of the loop regions and a short helix could not be built, due to the absence of electron density maps. Electron density was not present for the following residue stretches 14–21, 156–180, and 187–197 of chain A and the stretches 14–19, 143–146, 157–180, and 186–198 of chain B. The AtNRP2 monomer structure is represented in Figure 4c.

Each monomer could be divided into two domains similar to ScNAP1 and AtNRP1; domain I, which is the dimerization domain and domain II which is the histone-interacting domain (Figure 4d). The domain I consist of long antiparallel α-helices of the two monomers arranged in a “tram-track” fashion, which is the characteristic feature of all the NAP family proteins. The domain II essentially consists of five short α-helices and four antiparallel β-strands forming a β-sheet. The N-terminal dimerization helix is made up of residues 14–73, where the dimerization of the helix from the two monomers gets mediated through hydrophobic interactions between the two helices. The distribution of 18hydrophobic residues across the length of the helix stabilizes the dimer formation. The hydrophobic residues contributing to dimer formation are A21, L23, V24, L25, I28, L30, I33, L37, I40, A44, V48, L49, V51, V57, I58, V62, I69, I70, and I73 (Figure 4c). The β-sheet in domain II is formed by residues (103–150) and the five α-helices by α2 (76–83), α3 (85–91), α4 (92–101), α5 (202–212), and α6 (215–219). This kind of distribution of α-β-α arrangement of residues forms a three-layered structure, very similar to the other known NAP family proteins.

**Figure 5:**
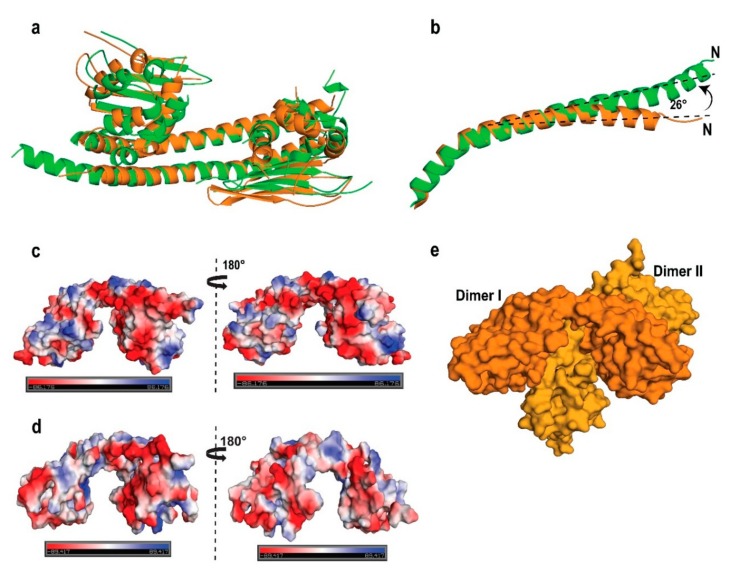
Comparison of AtNRP2 and AtNRP1 structures. (**a**) Superposition of the crystal structures of AtNRP1 (PDB id: 5DAY; Green) and AtNRP2 (Orange). The r.m.s.d. value observed for the structural superposition was 3.24 Å for 323 Cα atoms. (**b**) Representation of the superimposed dimerization helix of AtNRP2 and AtNRP1 monomers, revealing the shift in the helix. (**c**) Surface electrostatic potential of AtNRP1 in two orientations, representing electronegative (red), electropositive (blue) and neutral (white) areas. (**d**) Surface electrostatic potential of AtNRP2 representing electronegative (red), electropositive (blue), and neutral (white) areas. (**e**) The surface representation of AtNRP2 tetramer obtained by generation of symmetry mates.

The dome-shaped structure of AtNRP2 shows an uneven distribution of charge, where the concave face is more acidic in comparison to the convex face of the dimer (Figure 5d). The large negatively-charged underside region of the earmuff domain hints towards its importance in histone-binding. The charged tail regions which are absent in the crystal structure might also contribute to strengthening the histone interactions.

### 2.5. AtNRP2 Structure vs. AtNRP1 Structure

To gain more insights into the structure-function relationship of AtNRP2, we examined the structural differences of AtNRP2 with AtNRP1. Despite the overall domain organization being similar, we observed interesting structural differences between the two, which might have some functional relevance. We observed a relatively high root mean square deviation r.m.s.d. value 3.24 Å for 323 Cα atoms when AtNRP2 dimer was superimposed over that of AtNRP1 (Figure 5a). A relatively higher r.m.s.d. value for the two proteins which share 87% sequence identity suggested local structural differences. The long dimerization helix (α1) in AtNRP1 is slightly longer (58 residues) than AtNRP2 (51 residues). In addition, there exists an offset of 26° at N-terminal end of dimerization helix of AtNRP1 in comparison to that of AtNRP2 (Figure 5b). The offset in the dimerization domain leads to differences in the end-to-end distance of the curved cleft, and thus AtNRP2 is more curved than AtNRP1. In addition, we see an offset of around 4.4 Å in the relative position of the β-sheet of the earmuff domain of AtNRP2 in comparison to AtNRP1, which could also be attributed to the offset present in the dimerization helix. When the domain II of the two proteins was superposed, we got an r.m.s.d. value of 0.84 Å for 99 Cα atoms, indicating that the domain structure is similar and that the high r.m.s.d. while aligning the whole structure is primarily due to the offset in the dimerization domain. The offset present in β-sheet of the earmuff domain and the curved α1 helix together brings the two earmuff domains slightly closer in AtNRP2. The α3-helix is longer in AtNRP2 and is on a different plane as compared to AtNRP1. Another noticeable difference is in the loop connecting β4 and α5, where AtNRP1 contains a 3_10_-helix, which is absent in AtNRP2.

We observed another striking difference upon generation of AtNRP2 symmetry mates. AtNRP2 forms a tetramer assembly with its nearest neighbor (Figure 5e). Each tetramer is an oblate-shaped structure, having two dimers interlocked with each other via their concave faces, as opposed to the ring-like symmetry observed for the tetramer of VPS75, another NAP family member [44]. We also checked for the most stable assemblies possible for AtNRP2, using PDBePISA server [45], which suggested that stable tetrameric assemblies could also exist, along with the dimeric forms in solution (Figure 5e and Supplementary Table S3). This feature could be much relevant for the full-length AtNRP2, which we found to exist as a tetramer too. The underside of the earmuff domain is thought to contribute to histone interaction, and as the same region is blocked in the tetramer conformation, it appears that the tetramer assembly (if any) would occlude histone-binding or could probably be a means to regulate histone interaction. When we generated symmetry mates for the crystal structure of AtNRP1 dimer (PDB id: 5DAY), it did not reveal a tetrameric organization, perhaps because the crystal packing is very different. The most stable conformation in solution according to PDBePISA server also suggested AtNRP1 as a dimer, and hinted no possibilities for the existence of a tetramer, as it is the crystal symmetry that would be used for the prediction.

### 2.6. Structural Comparison of AtNRP2 with Other NAP Family Proteins

**Figure 6:**
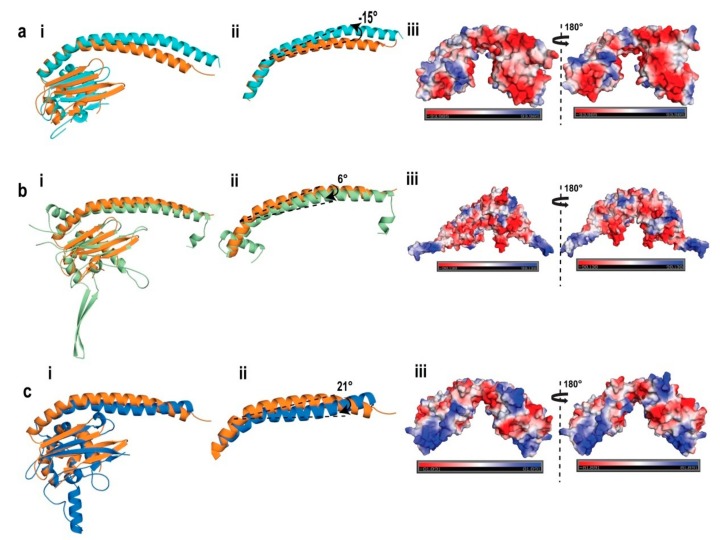
Comparison of AtNRP2 structure with the structure of other NAP family proteins. (**a**) (i) Structure superposition of AtNRP2 (orange) with HsSET/TAF-1β (PDB id: 2E50; cyan) r.m.s.d. = 4.94 Å for 304 Cα atoms. (ii) Figure highlighting the offset in the dimerization helix of AtNRP2 with HsSET/TAF-1β. (iii) Surface electrostatic potential of HsSET/TAF-1β in two orientations. (**b**) (i) Structure superposition of AtNRP2 (orange) with ScNAP1 (PDB id: 2Z2R; green) r.m.s.d. = 2.49 Å for 268 Cα atoms. (ii) Figure highlighting the offset in the dimerization helix of AtNRP2 with ScNAP1 (iii) Surface electrostatic potential of ScNAP1 in two orientations. (**c**) (i) Structure superposition of AtNRP2 (orange) with ScVPS75 (PDB id: 3DM7; blue) r.m.s.d. = 3.36 Å for 208 Cα atoms. (ii) Figure highlighting the offset in the dimerization helix of AtNRP2 with ScVPS75. (iii) Surface electrostatic potential of ScVPS75 in two orientations.

The structural differences of AtNRP2 with other proteins of the NAP family, such as ScNAP1, ScVPS75, and HsSET/TAF-1β were examined. ScNAP1 is the first structure to be studied from the NAP family [9]. Crystal structures are also available for HsSET/TAF-1β and ScVPS75 [18,40,46]. AtNRP2 aligned with the structures of HsSET/TAF-1β (PDB id: 2E50), ScNAP1 (PDB id: 2Z2R), and ScVPS75 (PDB id: 3DM7) with r.m.s.d. values of 4.94 Å for 304 Cα atoms, 2.49 Å for 268 Cα atoms and 3.36 Å for 208 Cα atoms, respectively (Figure 6). HsSET/TAF-1β, ScNAP1, and ScVPS75 are only 42.7%, 28.3% and 25.2% identical, respectively with AtNRP2. The structural alignment of all the three proteins with AtNRP2 revealed that the overall domain organization is very similar in all the proteins, wherein there is a long α-helical dimerization domain (domain I), followed by a protein interaction domain (domain II), containing both α-helices and β-sheets.

A short α-helix precedes the dimerization helix for ScNAP1, whereas, for HsSET/TAF-1β, ScVPS75, and AtNRP2 structures, the long dimerization helix forms the first helix (α1). The dimerization helices of all the NAP family structures take up an antiparallel “tram-track” fold rather than a coiled-coil motif, via strong hydrophobic interactions between the two helices. The length of the dimerization helices varies among all the different NAP family proteins, which is around 77 residue-long in HsSET/TAF-1β, 47 residue-long in ScNAP1, and 41 residue-long in ScVPS75, as compared to the 51 residue-long dimerization helix in AtNRP2. The end-to-end distance in decreasing order for the dome-shaped dimerization domain in different proteins is 82.5 Å for HsSET/TAF-1β, 77.6 Å for AtNRP2, 69 Å for ScNAP1, and 61.6 Å for ScVPS75. The structures with a shorter end-to-end distance are more curved and thus seem to have a tighter packing of the two helices. Due to the differences in the overall architecture of the dome formed by the dimerization helices, shifts of around −15°, 6° and 21° from that of AtNRP2 was observed for HsSET/TAF-1β, ScNAP1, and ScVps75, respectively (Figure 6). Importantly, the shift in HsSET/TAF-1β is in a curve-loosening direction (Figure 6a ii), while the shifts in ScNAP1 and VPS75 are in a curve tightening direction (Figure 6b,c ii). In all NAP family members, the dimerization helix shows a dome-shaped architecture, primarily due to certain specific residues within the helix. In ScNAP1, P131 and V109 provide a kink of around 30°and 25°, respectively to the helix. These two kinks in the structure of the dimerization domain allow the proper packing of the two helices together. The Pro residue is highly conserved among all the NAP family proteins, and it corresponds to P61, P65, P42, and P66 in AtNRP2, AtNRP1, ScVPS75, and HsSET/TAF-1β, respectively. The residue corresponding to V109 is replaced by other hydrophobic residues in all the proteins, except ScVPS75 where it is a charged residue. It is I40 in AtNRP2, I44 inAtNRP1, L45 in SET/TAF-1β, and K20 in ScVPS75. The overall distribution of hydrophobic residues along the length of the dimerization helix and the helical bending might result in a difference in the packing of the two monomers and hence the overall stability of the dimers. In general, the compact packing of these hydrophobic residues among the members of NAP family make the dimers highly stable and those could not be disrupted without denaturation [9]. In addition to the dimerization helix, ScNAP1 contains an extra alpha-helix in the N-terminus (α1) and another one (α3) after the long helix (α2). The helix α3 forms an accessory domain in the structure of ScNAP1.

The overall architecture of domain II is also quite similar in the case of all the NAP family proteins, except for the length of the α-helices and the β-sheets. All of them contain six α-helices and four β-strands, with the β-strands lying above the α-helices. In the case of HsSET/TAF-1β, the β-sheet presents an offset of 6.6 Å as compared to AtNRP2, which is due to a different trajectory caused by the offset in the long dimerization helix of HsSET/TAF-1β. A 46° shift was also observed for α3, in comparison to AtNRP2. The higher r.m.s.d. values are thus due to the shift in the position of the helices and strands. The differences in overall structure also suggest that the residues involved in histone-binding in the case of HsSET/TAF-1β could be different from those of AtNRP2. In contrast to this, ScVPS75 structure possesses two 3_10_-helices between β1-β2 and β4-β5, which are absent in AtNRP2. The comparison of the surface electrostatic potential of all the above three proteins with NRP2 shows ScNAP1 to be highly charged, followed by HsSET/TAF-1β and ScVPS75 in comparison to AtNRP2. Additionally, the concave histone-binding cleft is highly negatively charged and thus might show much tighter histone-binding as compared to AtNRP2.

The variation in the overall structure, differences in the position of the suggested histone-binding residues, and the resultant surface charge distribution among the various NAP family proteins (Figure 6) may result in differences in histone interaction and the number of histones to which they could bind.

### 2.7. The In-Solution Architecture of AtNRP1 and AtNRP2

**Figure 7:**
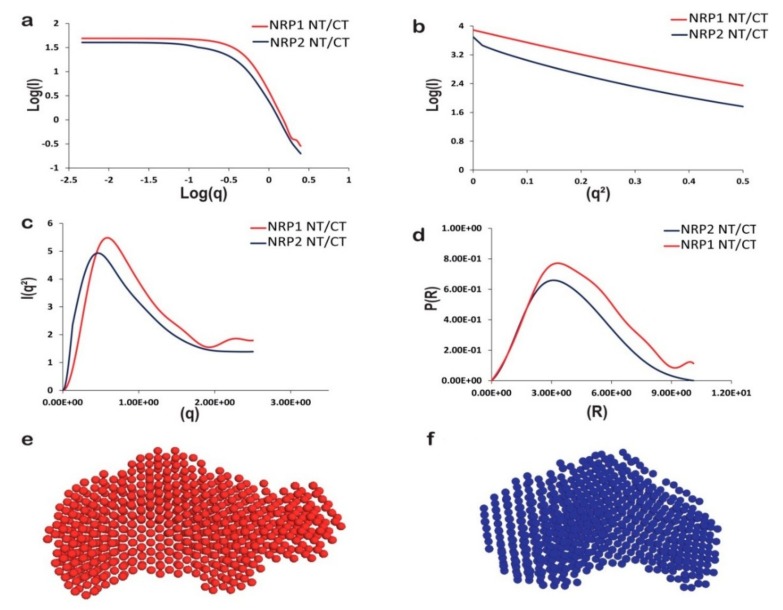
Small Angle X-ray Scattering (SAXS) profile and the generated envelope structures for AtNRP1 NT/CT and AtNRP2 NT/CT. (**a**) The double logarithmic plot for both AtNRP1 NT/CT and AtNRP2 NT/CT. (**b**) The Guinier plot analysis, showing the samples are monodispersed and homogeneous. (**c**) The Kratky plot showing the proteins to be elongated with flexible linker domains. (**d**) The Porod plot representing the pairwise distance distribution function. (**e**) The ab initio generated SAXS envelope of AtNRP1 NT/CT (red). (**f**) The ab initio generated SAXS envelope of AtNRP2 NT/CT (blue).

The crystal structures reveal the homodimeric forms of AtNRP1 [18] and AtNRP2. Since AtNRP2 NT/CT could form stable tetramers based on PDBePISA prediction, we carried out Small Angle X-ray Scattering (SAXS) experiments to gain more insights into its domain flexibility and confirm the in-solution oligomeric conformation. The SAXS experiments were performed with purified AtNRP2 NT/CT and AtNRP1 NT/CT. Since it was difficult to get a homogeneous sample for both AtNRP2 and AtNRP1 in full-length form. The data obtained from the SAXS experiments were analyzed using various programs from the ATSAS software suite [47] and the parameters such as the radius of gyration (Rg), the approximate molecular mass and the maximum size (D*_max_*) of the proteins were calculated. The SAXS plots and parameters obtained for AtNRP1 and AtNRP2 are provided in Figure 7 and Supplementary Table S4, respectively.For calculation of all the parameters, SAXS data were collected at three different concentrations. The scattering curves were comparable and did not show any concentration-dependent aggregation. The scattering curves were scaled and merged for further calculation. The effective size of the scattering particle (Rg) value calculated for both the particles was comparable and was estimated to be 3.02 and 3.25, respectively for AtNRP2 NT/CT and AtNRP1 NT/CT. The D_max_ value calculated was 9.77 Å for both AtNRP2 NT/CT and AtNRP1 NT/CT. These calculations suggest that AtNRP1 NT/CT is slightly bigger in dimension than AtNRP2 NT/CT. The Kratky plot analysis of both the proteins illustrated that the proteins are partially folded or they form a globular structure, along with a random-coil region (Figure 7c). It further suggests that both the proteins AtNRP2 NT/CT and AtNRP1 NT/CT have long flexible regions.

Further inspection of the P(r) curve showed both AtNRP2 NT/CT and AtNRP1 NT/CT to have an elongated shape (Figure 7d). Ab-initio modeling algorithm, DAMMIF [48] was used in combination with DAMAVER [49] to model both AtNRP2 NT/CT and AtNRP1 NT/CT. DAMMIF was used to generate 20 “dummy atoms” models, and further averaged with DAMAVER using normal mode analysis. The models selected for averaging of AtNRP2 and AtNRP1 (Figure 7e,f) were having NSD values of 0.914 and 0.933. The standard deviation of NSD was 0.043 and 0.071, and the models with a value higher than <NSD> + 2*SD were discarded.

The theoretically calculated SAXS curves for the models and the actual experimental data were in good agreement with each other, and the chi-square (χ^2^) values obtained were 0.905 and 0.858, respectively for AtNRP2 NT/CT and AtNRP1 NT/CT. The program CRYSOL [50] was used to check the fitting of the chosen model and was giving higher χ^2^ values for both the proteins. Further refinement using SREFLEX [51] brought the χ^2^ values to 9.3 for AtNRP2 NT/CT, but the χ^2^ values for AtNRP1 NT/CT could not be brought down at all. The higher values for both the structures suggest flexibility and greater domain movements in solution, in comparison to those observed in the crystal structures. We made use of the different programs present in ATSAS software to estimate the approximate molecular mass of both AtNRP1 NT/CT and AtNRP2 NT/CT (Supplementary Table S4). The estimated molecular masses were nearly similar to theoretical molecular mass, and thus both the proteins were confirmed to be predominantly dimer in solution. It is not surprising that we did not see higher oligomeric forms for the two proteins in SAXS, as the NT/CT proteins might exist predominantly as homodimers as was observed for AtNRP2 NT/CT in the sedimentation velocity analytical ultra-centrifugation experiment. A mixed population sample for the full-length proteins would not provide clean data from SAXS experiments, even if we wanted to try. Overall, the above findings illustrate that these proteins exist as elongated homodimers in solution, perhaps with a high degree of disorderedness and/or domain flexibility.

### 2.8. Stability of AtNRP1 and AtNRP2

Previously it was reported that a global disruption of secondary structure is required for separating ScNAP1 monomers from the dimer [9]. From our comparative analysis AtNRP2 structure with all the other known NAP family proteins, we have observed differences in the overall length of the dimerization helix, the distribution of hydrophobic residues and the overall packing of AtNRP2. Therefore, we wanted to check whether the differences translate to differences in the stability between AtNRP1 and AtNRP2. This may further give clues regarding the differences between AtNRP1 and AtNRP2 with respect to their function and relevance in the plant.

**Figure 8:**
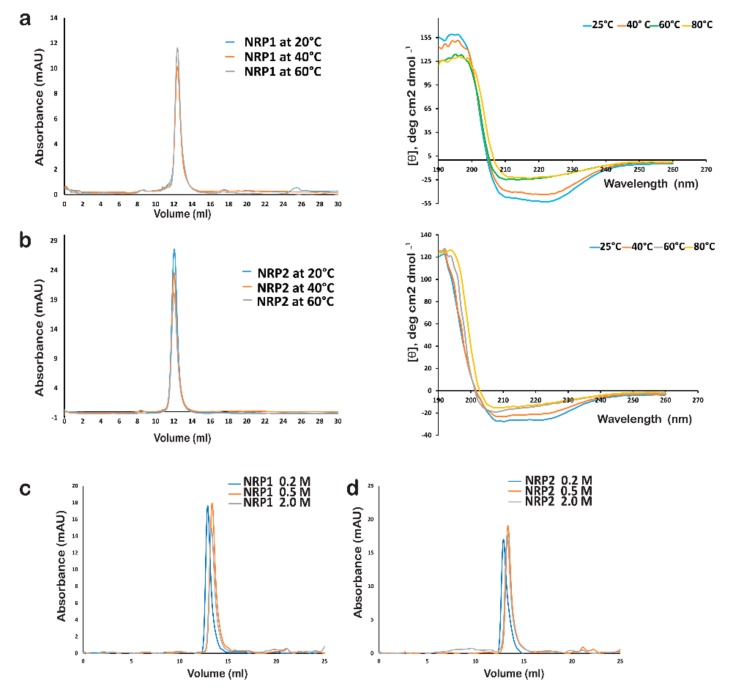
Analysis of AtNRP1 and AtNRP2 stability. Analytical gel-filtration chromatograms (left panel) and Far-UV CD curves (right panel) for the full-length forms of (**a**) AtNRP1, and (**b**) AtNRP2, showing the elution of the respective proteins at the same position, irrespective of heating at different temperatures and Far-UV CD curves for 25, 40, 60, and 80 °C respectively, showing no major change in secondary structure at the respective temperatures. Analytical gel-filtration chromatogram for the full-length forms of (**c**) AtNRP1, and (**d**) AtNRP2 showing the elution of the respective proteins at the same position, irrespective of the salt concentrations. All the gel-filtration experiments were carried out in a buffer containing 20 mM Tris-HCl (pH 8.0), but with different concentrations of NaCl (0.2, 0.5, and 2.0 M) using a Superdex 200 10/300 GLcolumn.

The thermal stability of AtNRP1 and AtNRP2 dimers was carried out by heating the full-length proteins at definite temperatures (20, 40, and 60 °C) followed by analytical gel-filtration chromatographic purification. The differences in stability could be monitored by the shifts in elution volume. The analytical gel-filtration chromatograms showed both the proteins to be stable until 60 °C, as no major shift was observed in the position of peaks of both AtNRP2 and AtNRP1. However, the peak height was decreasing with increase in the treatment temperature, suggesting the formation of mild soluble aggregates, which could be seen as a small peak in the void volume of chromatograms (Figure 8a,b). We also performed circular dichroism (CD) thermal melt analysis of full-length AtNRP2 and AtNRP1 to probe the possible differences in unfolding and the stability of the proteins. CD thermal melt analysis (Figure 8a,b) showed the protein to be stable until high temperatures, as complete unfolding was not observed in the CD curves, although we see a shift in the CD profiles at both 222 and 208 nm at 60 °C and higher temperatures. The thermal melting profile also revealed that the Tm values could be nearly similar for both the proteins. To further analyze the differences in thermal stability of both the proteins, we heated the proteins for a longer duration of 10 min and subjected it to analytical gel-filtration chromatography (Supplementary Figure S3a,b). The analytical gel-filtration chromatogram revealed AtNRP1 to have more aggregation at 60 °C compared to AtNRP2 at the same temperature. This could be due to the slightly tighter packing of AtNRP2 in comparison to AtNRP1.

Since we observed a longer hydrophobic stretch in the dimerization helix of both AtNRP2 and AtNRP1, we further characterized the stability of the two dimers at different salt concentrations. AtNRP1, AtNRP2, and their truncated forms were brought into buffers with salt concentrations of 0.2, 0.5, and 2.0 M NaCl, respectively by dialysis. The proteins were then subjected to analytical gel-filtration chromatography purification in the respective buffers with the same salt concentrations (Figure 8c,d). Though no difference was observed for the elution volumes of the truncated proteins with increasing salt concentrations, a minor shift was observed for the full-length proteins, indicating a smaller size or more compact shape with increasing salt concentrations. The minimal shift, however, does not attribute to the falling apart of dimers into monomeric forms. The full-length proteins with extended charged residues at the termini might be taking up a more compact structure in the presence of higher salt. The data suggested that both AtNRP1 and AtNRP2 are highly salt-stable, similar to ScNAP1 and this result supports the data obtained from crystal structures of both the proteins, where we found that extensively distributed hydrophobic residues stabilize the dimers over the two anti-parallel α-helices.

### 2.9. Histone-Binding and Chaperoning Activities of AtNRP1 and AtNRP2

The interaction studies of AtNRP1 with histones suggested a preferential binding with H2B over H3 [18,22]. The Kd values for the interaction of AtNRP1 with H2A-H2B and H3-H4 tetramer have been reported as 0.1 and 0.4 μM, respectively [22]. However, the binding of AtNRP2 with histones has not been tested previously. Herein, we have compared the histone dimer binding capabilities of AtNRP2 and AtNRP1.

**Figure 9:**
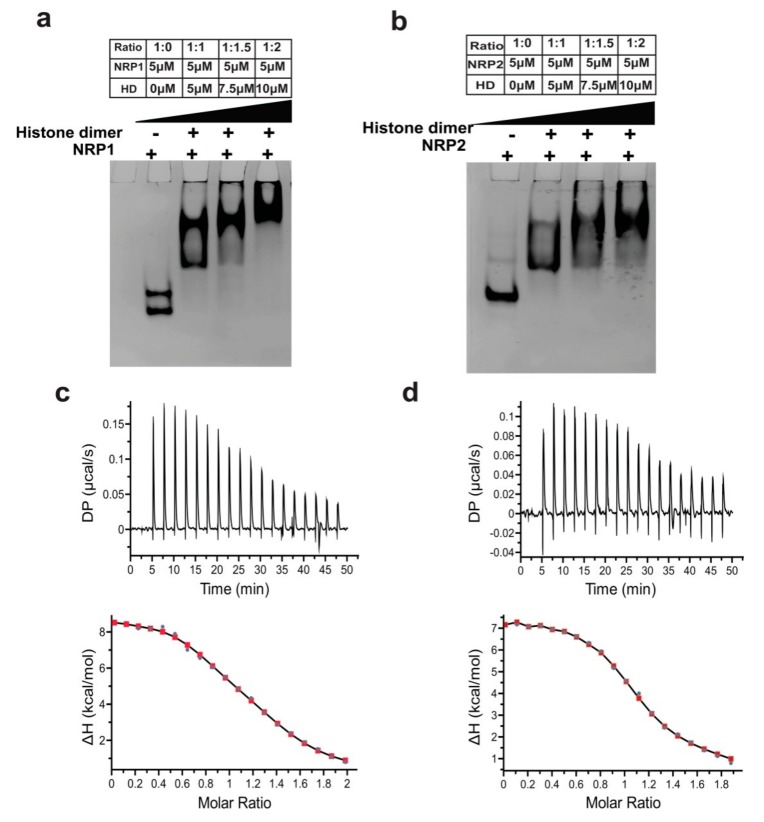
Interaction of AtNRP1/AtNRP2 with histone H2A-H2B dimer. Electrophoretic mobility shift assay for the binding of histone dimer with (**a**) full-length AtNRP1 and (**b**) full-length AtNRP2. (**c**) The binding isotherm of full-length AtNRP1: H2A-H2B. (**d**) The binding isotherm of full-length AtNRP2: H2A-H2B. The top panel represents the sequential injection of AtH2A-H2B into AtNRP1 and bottom represents integrated heat data after correction for the heat of dilution. The data fitting was done using sequential binding model. (Red rectangles represent experimental fit and grey circles represent actual fit).

Electrophoretic mobility shift assay (EMSA) was carried out to visualize the interaction of histone dimer with both AtNRP1 and AtNRP2 and their truncations on a native polyacrylamide gel. A band corresponding to a higher molecular weight was observed for the full-length AtNRP1 by itself and the same was not visible for the full-length AtNRP2 at 5 μM concentration. However, a similar higher molecular weight band for the full-length AtNRP2 was visible at higher concentrations (data not shown). Since the full-length AtNRP2 tend to form higher order oligomers, as has been seen from analytical ultra-centrifugation experiments, it could be the higher oligomeric form (perhaps, a tetramer) of the NRPs that appear at higher protein concentrations in EMSA, indicating a concentration-dependent oligomerization event of the proteins. However, this oligomerization tendency was absent in AtNRP1 NT/CT and AtNRP2 NT/CT samples, which showed no higher oligomers even in analytical ultra-centrifugation experiments. Increasing amounts of histone H2A-H2B dimer was titrated into a constant amount of AtNRP1/2 proteins (5 µM). The EMSA revealed distinct bands of the complex of AtNRP1/2 with H2A-H2B. The titration showed saturation at about 1:2 ratio for AtNRP1/2 dimer and the histone dimer (Figure 9a,b). However, the EMSA for AtNRP1 NT/CT and AtNRP2 NT/CT (Supplementary Figure S4a,b) did not show a saturation pattern similar to the full-length proteins. The truncated proteins, in fact, did not attain saturation. This suggested that the full-length proteins can bind stronger to the histones.

We also performed isothermal titration calorimetry to analyze the interaction of histone H2A-H2B with full-length AtNRP2 and AtNRP1 and to compare the thermodynamic parameters for their histone interaction (Table 2). The titration profile shows the endothermic heat of binding, which is indicative of the reaction being stabilized by entropic factors (Figure 9c,d). In such entropy-driven reactions, the release of an ordered water molecule from the binding site is quite likely to be a significant contributor. Since in our previous EMSA experiment, we observed a 1:2 binding ratio of NRP1 and NRP2 homodimer to histone dimer, we initially tried to fit our data using ‘two-set of binding sites’ model, but we could not achieve any proper fitting. Thus, we further tried to fit the data using ‘sequential binding model’ and achieved a proper fitting of the enthalpy curve against molar ratio. The Kd values (Kd_1_ and Kd_2_) obtained for AtNRP2 is lower than AtNRP1, which suggests that AtNRP2 has a stronger affinity for H2A-H2B. The sequential binding model corresponds to more than two ligand binding sites which could be either identical or independent or cooperative. From our results, we could only say that both the protein dimers have two histone-binding sites, wherein the one binding site has a much higher affinity for H2A-H2B, compared to the other.

**Figure 10:**
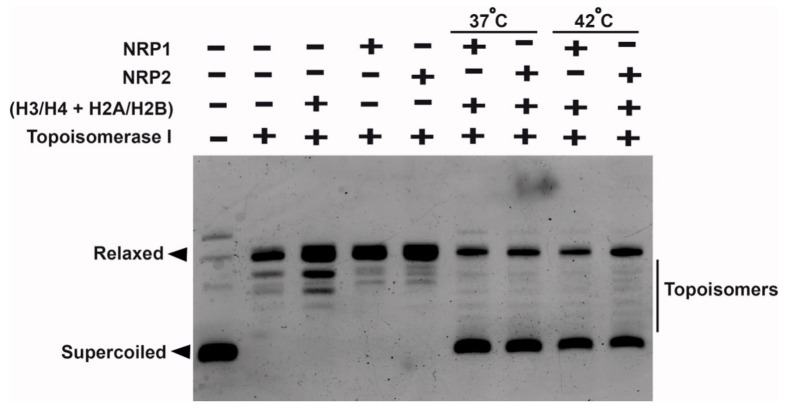
Plasmid DNA supercoiling assay. The plasmid DNA supercoiling activity was carried out for full-length forms of both AtNRP1 and AtNRP2. The samples were run on 1% agarose gel and stained with ethidium bromide. Relaxed and supercoiled forms of plasmids are labeled in the figure. Topoisomers are the plasmid DNA bands corresponding to different degrees of supercoiling, due to the effect of histone chaperones.

Next, to confirm the function of AtNRP2 and AtNRP1 as histone chaperones, we performed the classical plasmid DNA supercoiling assay. The more the supercoiling, the faster the migration in electrophoresis will be. The nucleosome formation in this assay is indirectly shown by the quicker electrophoretic migration, due to supercoiling of the relaxed plasmid DNA. Both AtNRP1 and AtNRP2 were capable of increasing plasmid supercoiling or nucleosome assembly in vitro at 37 °C. We observed a comparable degree of supercoiling by AtNRP1 and AtNRP2 in this assay and as such both could be equally functional as histone chaperones. Although we carried out the same in vitro assay at 42 °C, in order to check whether AtNRP2 could be more effective as a chaperone under a condition mimicking thermal stress, we did not see much difference between AtNRP1 and AtNRP2 in their ability to aid nucleosome assembly (Figure 10). However, the nucleosome assembly process within the plant nucleus will be dependent on several other factors, along with the histone chaperones.

## 3. Discussion

The packaging of eukaryotic chromatin and assembly of nucleosome have been driving essential questions by chromatin biologists in the last few decades. The discovery of histone chaperones and their ability to assemble nucleosomes in vitro [52] have led to studies on their structure and function. Nucleoplasmins and NAP1 form the two well-characterized and extensively studied histone chaperones. Both of them are H2A-H2B specific histone chaperones [6,53]. The exchange of the histone H2A-H2B dimer is crucial for controlling nucleosome dynamics, making studies on H2A-H2B interacting histone chaperones very important. The crystal structure of ScNAP1 bound to H2A-H2B has elucidated the mechanism underlying NAP1-mediated histone chaperoning and nucleosome assembly [54]. Unlike human and yeast histone chaperones, it is still unclear regarding how the different plant histone chaperones orchestrate the process of nucleosome assembly and disassembly [55] and whether they perform any accessory functions as well. NAP family proteins are much more divergent in plants; wherein, *Arabidopsis* and rice plant both have four NAP1 isoforms and two NRPs each [5,12,17,18]. The structure of AtNRP1 has been the only one from the plant NAP protein family [18]. Apart from NRP1 structure, only one more plant histone chaperone structure is known, i.e., Spt16N-domain of FACT complex from *Cicer arietinum* [56] is available. Thus, structural studies on plant histone chaperones require more attention.

AtNRP2 is a homolog of AtNRP1 and is 87% identical to AtNRP1. The studies done on single and double *nrp*-mutants have shown differential expression of a greater number of genes in the *nrp2-1* mutant than both *nrp1-1* mutant and *nrp1-1 nrp2-1* double mutant [5]. The two proteins show a similar expression pattern, but still seem to have considerable differences in their function, which prompted us to ask a question of why AtNRP2 is then needed in the plant. Our phylogenetic analysis revealed that the presence of *nrp1* and *nrp2* genes is not a standard feature among all the plants. Instead, most of the plants possess only NRP2, and very few of them possesses both NRP1 and NRP2. Thus, it seems that NRP2 is more critical for the plant than NRP1. Another interesting finding is that in *A. thaliana* itself, the two genes have evolved independent of each other, by gene duplication. Non-redundant database search and blast analysis revealed that NRPs are present extensively in dicot plants, and only very few monocots are found to possess NRP or similar proteins. As such, the evolution of histone chaperones also seems associated with the divergence and evolution of monocots and dicots [14]. It has been suggested earlier that the various plant histone chaperone families have multiple members due to some events of segmental duplication, leading to the expansion of the gene families. Based on phylogenetic analysis it has also been shown that *A. thaliana* NRP clusters differently from the classic NAP domain-containing proteins, suggesting them to possibly have a different physiological function than the NAP1 class proteins [12,14,53]. NRPs have previously been shown to be significant for root development in plants [5,18]. Therefore, the overall architectural differences in roots of dicots and monocots could also be one of the reasons for the differences in the expression pattern of *nrp* genes within these plants. Our analysis showed that *Amborella trichopoda* NRP is nearest to that of monocots, and hence studies on this protein might provide us significant clues about the differences in structure and function of NRPs in dicots and monocots. As these histone chaperones are known to interact with different transcription factors, the presence of both NRP1 and NRP2 in a single plant might also have a justification beyond redundancy [53]. The plant transcription factor WEREWOLF (WER) interacts with AtNRP1 at *GLABRA2* promoter and helps in the eviction of histones, leading to gene activation in the root cells [18]. The possible interaction between WER and AtNRP2 also need to be studied to understand whether the two NRPs have differential binding preferences for WER. Additionally, the studies on transcription factors and their interaction with NRPs during different stress conditions could provide significant insights into the actual function of these proteins.

Further, the biophysical and structural characterization of AtNRP2 shows it to exist as a homodimer in solution, with extensively flexible termini. The distribution pattern of hydrophobic residues in the dimerization helix is another remarkable feature of the NRPs. Though we were unable to crystallize the full-length protein, our biophysical studies suggest that the flexible termini could be of prime importance in deciding the oligomerization properties of both the NRPs and their interaction strength with partner proteins. The possible post-translational modifications in these unstructured regions and their effect on interactions with other proteins also need to be addressed. AtNRP2 contain a typical NAP domain and has a “headphone fold” similar to AtNRP1, SET/TAF-1β, and the other NAP family proteins. In the case of HsSET/TAF-1β, the gene is repressed upon interaction with the transcription factor Sp1. However, AtNRP1 interaction with the transcription factor WER at GL2 promoter leads to activation of gene expression [18,57]. This difference in activity again shows that despite having similar domain architecture, the NAP family proteins may perform different functions depending on their interaction with specific families of transcription factors. It will not be surprising if AtNRP2 shows differences with AtNRP1 in its binding affinity with the interacting transcription factors or even may interact with an entirely different set of transcription factors.

We observed both AtNRP1 and AtNRP2 to be highly stable in buffers with very high ionic strength; as high as 2.0 M NaCl. This feature has been observed for ScNAP1 as well [9]. The higher stability and the unique distribution of hydrophobic residues over the dimerization interface led us to test the thermal stability of the two proteins. In our analytical gel-filtration chromatography experiments, after heating to various temperatures, both AtNRP1 and AtNRP2 were found to be stable until 42 °C. AtNRP1 heated to 60 for 10 min showed signs of aggregation, whereas AtNRP2 heated to 60 °C was still stable and did not aggregate. However, thermal stability measurement by CD-spectroscopy shows both of them to be stable until 60 °C, which could be possible because of the shorter heating time used during the thermal ramping experiment. Previously, AtNRP1 has been reported to increase the survival rate of seedlings and cells exposed to 45 °C for one hour [20]. Thus, our data showing higher thermal stability might suggest that at higher temperatures, these proteins may be more relevant as chaperones, wherein perhaps, AtNRP2 has a more significant role to play.

The differences in the surface charge distribution and the overall architecture of the dimerization domain seem to be crucial in histone-binding abilities of the various NAP family proteins. The differences in structure also suggest why NAP1-related proteins (NRPs) might also be required in addition to NAP1 proteins in the different organisms. A comparison of expression and binding abilities of NAP and NRP during biotic and abiotic stress conditions is required for a better understanding of the functions of these proteins and possible redundancy. Moreover, NRP proteins are known to be a nucleo-cytoplasmic protein which harbors only NLS [53]. The absence of a nuclear export signal (NES) and accessory domains in NRP structures in comparison to ScNAP1 are also striking features. ScNAP1 is known to perform nucleo-cytoplasmic shuttling, but NRPs are completely nuclear localized. The NRPs thus might perform particular functions in comparison to NAPs due to their specific sub-cellular localization.

Mobility shift assays with AtNRPs and histone dimers showed that both the proteins can interact with histone dimers in vitro, but the presence of their tail regions, especially the C-terminal tail is essential for tighter interaction with histone oligomers. The comparison of the strength of binding by the ITC experiment helped us to understand the direct interaction of these proteins with histone dimer and their role as an H2A-H2B histone chaperone. The ITC data showed that both AtNRP2 and AtNRP1 have more than one binding site which could be identical or non-identical. EMSA experiment also revealed a 1:2 binding stoichiometry for NRP dimers with H2A-H2B dimer, similar to a previous report for ScNap1 [3]. AtNRP2 revealed a higher binding affinity to H2A-H2B as compared to AtNRP1 which again suggests the importance of AtNRP2 in plants. Strikingly, we observed a unique tetrameric form of AtNRP2 by generating symmetry mates of its crystal structure, where the histone-interacting stretch of the two dimers gets involved in tetramer formation. Therefore, it could be possible that during histone transport, the tetramer dissociates into dimers and each dimer would then associate with histones. This kind of arrangement, though possible for AtNRP1 also, was not observed perhaps, due to differences in AtNRP1 packing in the crystal lattice. The DNA supercoiling assay further revealed that both AtNRP2 and AtNRP1 are capable of nucleosome assembly in vitro, which supports their function as histone chaperones. The increased stability, stronger H2A-H2B interaction potential, and the ability to assemble nucleosomes show the relevance of AtNRP2, perhaps, as a histone chaperone with a significant role during stress conditions. However, its function as a stress-related histone chaperone needs to be further validated in vivo.

More recently it has been shown that AtNRP1 shows a competitive mode of binding with Cytochrome C and histone dimers [22]. Since the two proteins, AtNRP2 and AtNRP1 share a similar structural fold, Cytochrome C might interact with AtNRP2 as well. A comparative study of Cytochrome C interaction with AtNRP1 and AtNRP2 may improve our understanding of the role of these proteins during DNA damaging stress conditions. In addition, the role of NRPs in the regulation of transcription and its interaction with transcriptional activators and repressors needs to be focused further to improve our understanding of their specific roles.

## 4. Materials and Methods

### 4.1. Phylogenetic Analysis

The phylogenetic analysis and tree construction was performed by MEGA 7.0 software suite [25]. The protein sequences were obtained from NCBI, using AtNRP2 sequence (accession no. NP_564063) as a query and protein BLAST search was done against non-redundant protein sequences with a maximum of 250 targets. The sequence alignment was done using MUSCLE program version 3.8 (Mill Valley, CA, USA) [24] from MEGA 7.0. Gene duplications were identified by searching for all branching points in the topology, with at least one species that is present in both subtrees of the branching point. An unrooted gene tree was used for the analysis, such that the search for duplication events was performed by finding the placement of the root on a branch or branches that produced the minimum number of duplication events. Additionally, aPhylome DB search [29] was done using AtNRP2 as seed sequence, and a BLAST search was carried out. Hit Id: Phy00018Y8_ARATH, showed 100% identity and 85.94% overlap with AtNRP2 protein sequence, and a phylogenetic tree was generated using this hit.

### 4.2. Construction of E. coli Expression Plasmids, AtNRP1/AtNRP2 Expression, and Purification

The individual open reading frames ORFs coding for full-length AtNRP1, and AtNRP2, optimized for bacterial expression were obtained from Genscript (Piscataway, NJ, USA) in a pET22b(+) plasmid between NdeI and XhoI sites, for expression with non-cleavable C-terminal hexa-histidine tag. The ORFs coding for the N and C-terminal truncated versions AtNRP1_14-226_ and AtNRP2_14-223_ were prepared from these constructs and cloned into a pGEX-6P-1 plasmid between BamHI and SalI sites, for expression with an N-terminal cleavable GST-tag. All the recombinant proteins were overexpressed in *E. coli* BL21 (DE3) cells at 37 °C for 3 h in the 2xYT medium by inducing the culture grown to an OD_600_ of 0.6 with 0.6 mM IPTG.

The cell pellets after overexpression of the full-length proteins with His-tag was washed as described in a previous protocol [58]. The cells were then lysed in buffer A (20 mM Tris-HCl (pH 8.0), 750 mM KCl, 1 mM MgCl_2_, 10% glycerol, 10 mM Imidazole, 1 mM PMSF, and 2 mM 2-mercaptoethanol) using a cell homogenizer. The cell lysate supernatant (after centrifugation at 42,000× *g* for 90 min) was passed through a HisTrap FF 5 mL nickel affinity column (GE Healthcare, Chicago, Illinois, USA) pre-equilibrated with the buffer A and then washed with buffer B (20 mM Tris-HCl (pH 8.0), 500 mM KCl, 1 mM MgCl_2_, 25 mM Imidazole and 2 mM 2-mercaptoethanol), followed by a gradient elution using an elution buffer (20 mM Tris-HCl (pH 8.0), 500 mM KCl, 1 mM MgCl_2_, 300 mM Imidazole (pH 8.0), and 2 mM 2-mercaptoethanol). The fractions from affinity purification was further purified by gel-filtration chromatography using a Hiload 16/600 Superdex 200pg column (GE Healthcare) in SEC buffer (20 mM Tris-HCl (pH 8.0), 200 mM NaCl, 1 mM EDTA, 1 mM PMSF and 1 mM 2-mercaptoethanol). The chromatographic purifications were carried out with the help of an ÄKTA Pure 25 M chromatography system (GE Healthcare) housed in a cold cabinet and maintained at 4 °C.

The cell pellet after overexpression of the full-length proteins with GST-tag was lysed into a GST lysis buffer (50 mM Tris-HCl (pH 7.5), 2 mM EDTA, 500 mM NaCl, 1 mM PMSF, 10% glycerol, and 1 mM 2-mercaptoethanol) using a cell homogenizer and then the lysate was warmed at 40 °C for 10 min in a water bath. The cell lysate was then centrifuged at 42,000× *g* for 90 min, and then the supernatant obtained was passed through GSTrap FF 5 mL column (GE Healthcare) using a peristaltic pump. The sample was allowed to bind for 16 h by continuous recirculation of the sample through the column and then washing was done with a low salt wash buffer (20 mM Tris-HCl (pH 7.5), 2 mM EDTA, 150 mM NaCl, and 1 mM 2-mercaptoethanol] and a high salt wash buffer [20 mM Tris-HCl (pH 7.5), 2 mM EDTA, 900 mM NaCl, and 1 mM 2-mercaptoethanol), followed by another round of wash with the low salt wash buffer. The GST tag was cleaved on-column with the help of PreScission protease (GE Healthcare). The cleaved protein after removing GST-tag was then collected from the flow-through and wash fractions from the column. The protein after affinity purification and GST-tag cleavage was further purified by gel-filtration chromatography using a Hiload 16/600 Superdex 75pg column (GE Healthcare). All the protein samples were analyzed on 15% SDS-PAGE and were visualized by Coomassie brilliant blue staining.

### 4.3. Histone Expression and Purification

For histone H2A-H2B dimer preparation, a co-expression construct for codon-optimized ORFs of *Arabidopsis thaliana* H2A and H2B cloned into the pCDFDuet-1 vector (Merck KGaA, Darmstadt, Germany) was obtained from Genscript. In this construct, the ORF coding for AtH2A was cloned between BamHI and EcoRI sites in the MCS-1 and the ORF coding for AtH2B was cloned between NdeI and BglII sites in the MCS-2 of the vector. The construct was transformed into *E. coli* Lemo21 DE3 cells (New England Biolabs, Ipswich, MA, USA). The co-expression of histones was carried out for 3 h, at 37 °C with 0.6 mM IPTG, when the culture grown in 2xYT medium reached an OD_600_ of 0.6. The cells after overexpression were harvested by centrifugation, lysed using a cell homogenizer and purified by His-tag affinity purification similar to full-length AtNRP1/2. The protein was further purified by gel-filtration chromatography in a 20 mM Tris-HCl (pH 8.0) and 2 M NaCl buffer using Hiload 16/600 Superdex 200pg column.

### 4.4. Crystallization and Data Collection

Single AtNRP2, NT/CT crystals, were obtained in a condition 27 of AmSO4 Suite (Qiagen, Venlo, Netherlands) having 0.2 M potassium fluoride and 2.2 M ammonium sulfate. The crystals for diffraction studies were transferred into the same condition supplemented with 20% glycerol and flash cooled in liquid nitrogen. A total of 2000 frames with 0.10°oscillation were collected at a wavelength of 0.9794 Å from the ESRF Grenoble beam line ID30A-3 at a resolution of 3.4 Å. The diffraction data were processed using XDS [59]. The crystal belonged to H32 space group, with unit cell dimensions a = 123.45 Å, b = 123.45 Å, c = 228.71 Å, α = 90°, β = 90°, and γ = 120°. Two molecules, indicative of a dimer were present in an asymmetric unit.

### 4.5. Structure Determination

The crystal structure of AtNRP2 NT/CT was solved by molecular replacement method with the program Molrep [60] from CCP4 program suite [61], using the coordinates of AtNRP1 crystal structure (PDB id-5DAY) as a search model. The refinement and model building were carried out by programs such as Refmac [62], Phenix Refine [63,64], and COOT [65]. The final model was checked for stereochemical quality and Ramachandran plot by Molprobity [66], and surface analysis was carried out using PISA [67]. The structure figures were prepared with the help of the PyMOL molecular graphics system (Schrodinger, LLC, Cambridge, MA, USA).

### 4.6. Analytical Gel-Filtration Chromatography

Analytical gel filtration chromatography was done with a Superdex 200 10/300 GL column (GE Healthcare). A total of 100 µL of each of the protein samples were injected and allowed to pass through the column at 4 °C with 0.5 mL/min flow rate in a buffer containing 20 mM Tris-HCl (pH 7.5) and 150 mM NaCl. The standard proteins from the gel-filtration high molecular weight calibration kit (GE Healthcare) were passed through the column in the same buffer at 4 °C to aid molecular weight estimation. For analyzing the stability at different salt concentrations, the similar buffer was used, but with varying NaCl concentrations. For thermal stability analysis, the samples were heated at 25, 42, and 60 °C, respectively, followed by a spin at 13,000× *g* for 10 min at 4 °C, before subjecting to gel-filtration, also at 4 °C.

### 4.7. Sedimentation Velocity Analytical Ultra-Centrifugation

Sedimentation velocity analytical ultra-centrifugation experiment was done using a ProteomeLab XL-A centrifuge (Beckman Coulter, Brea, California, USA) equipped with An-50 Ti analytical 8-place titanium rotor. The sectors of each Epon double-sector centerpieces were filled with 400 µL of SEC buffer and 390 µL of the protein sample, respectively. The AtNRP2 and AtNRP2NT/CT samples were diluted in their SEC buffer to obtain an OD_280_ of 0.5. The samples were centrifuged at 40,000 rpm, at a temperature of 20 °C and 350 frames were collected. Absorbance data were acquired at a wavelength of 280 nm by taking two averages per scan. Absorbance scans were taken at an interval of 1 min. Buffer viscosity, protein partial specific volume, and viscosity were calculated using the software SEDFIT [68], and data analysis were performed with SEDFIT [68] and SEDPHAT using c(s) distribution analysis [69]. C (s) distribution plots were made using GUSSI.

### 4.8. Circular Dichroism Spectroscopy

The near-UV CD spectrum was measured using a Circular Dichroism spectropolarimeter (Applied Photophysics, Leatherhead, Surrey, UK). The protein samples were brought into 1xPBS by diluting 5 µL of protein (1 mg/mL) in 995 µL of 1xPBS buffer. For thermal ramping experiments, the samples were heated gradually from 25 to 90 °C in the CD spectropolarimeter while taking measurements at a ramp rate of 1 °C per minute. To monitor changes in the CD spectra as a function of increasing temperature, the ellipticity (θ) in mill degrees was plotted against temperature values for each 10 °C interval. Three consecutive scans were accumulated, and average spectra were used.

### 4.9. Small Angle X-ray Scattering Analysis

The Small Angle X-ray Scattering (SAXS) experiments were performed at ESRF beam line BM29. The SAXS data were collected at sample concentrations of 5, 3, and 2 mg/mL, respectively. The frames were collected with 100% beam intensity and at an exposure of 1 s with an X-ray wavelength of 0.9919 Å. The data was recorded on the Pilatus 1M detector; the detector distance was 2.87 m, and the exposure temperature was 10 °C. Integration and buffer subtraction were done by auto-processing software available at the beam line. The data analysis and modeling were done by ATSAS 2.8 [47]. To obtain necessary structural information and to judge the quality of SAXS data, we first performed an analysis of the one-dimensional SAXS experimental curves. The Guinier analysis was done by the program Primusqt [70] from ATSAS. The radius of gyration (Rg) was calculated from the slope of the Guinier plot. The quality of data for both the samples was evaluated by the linear plot obtained from Guinier analysis. The approximate molecular mass of the protein was also calculated from the SAXS curve analysis. The maximum size of the protein (D*_max_*) was calculated using Pair-Distance Distribution function [P(r)] by trial and error procedure. All the parameters were calculated using the program GNOM [71]. Ab initio modeling was done by DAMMIF [48], and the average model was collected from 20 runs using DAMMAVER [49]. The molecular weight was estimated using the ATSAS analysis tool for molecular weight calculation.

### 4.10. Isothermal Titration Calorimetry

The isothermal titration calorimetry was performed using MicroCal PEAQ-ITC (Malvern Instruments, Malvern, Worcestershire, UK) at 25 °C. The purified *A. thaliana* H2A-H2B was taken in the syringe at 200–210 μM concentration, and AtNRP1/AtNRP2 was taken in the sample cell at 20 μM concentration. Before the titration experiment, all the protein samples were dialyzed extensively against a buffer containing 20 mM PIPES (pH 7.4) and 300 mM NaCl. The reference cell was filled with distilled water. A total of 19 injections were given with 2 μL volume per injection, except for the first injection and 150-s interval was given between two consecutive injections. The data were normalized using a composite model where ligand into the buffer, and buffer-buffer titration were subtracted from the original data. The fitting was done using the sequential binding site model. All the analysis was done using MicroCal PEAQ-ITC Analysis software version 1.0 (Malvern Instruments).

### 4.11. DNA Supercoiling Assay

The nucleosome assembly activity of AtNRP1 and AtNRP2 protein was done based on the protocol described previously with slight modifications [72,73]. To obtain relaxed and closed circular DNA, 0.5 μg of negatively supercoiled pUC19 (2,686 bp) plasmid was pre-incubated with Wheat Germ Topoisomerase1 (1 unit per μg of DNA; Inspiralis) in a reaction volume of 30 μL at 37 °C for 40 min. Separately, the histones H2A-H2B and H3-H4 (1.0 μg each) were pre-incubated with either AtNRP1 and AtNRP2 (0.5 μg) at 30 °C for 30 min in 50 μL reaction buffer containing 20 mM Tris-HCl (pH 7.5), 0.10 mM NaCl,1.0 mM MgCl_2_, 0.5 mM DTT, and 0.1 mg/mL BSA. The pre-treated plasmid and the histone chaperone reaction mixture were added together and further incubated at 37 °C and 42 °C for 90 min. The reaction was stopped by adding an equal volume of solution containing 20 mM EDTA, 1% *w/v* SDS and 0.2 mg/mL proteinase K, and incubating at 37 °C for 30 min. After stopping the reaction, the plasmid sample was extracted using phenol-chloroform treatment and precipitated with ethanol. The obtained plasmid DNA was resolved by 1% agarose gel electrophoresis followed by ethidium bromide staining.

## Figures and Tables

**Table 1:** Data processing and refinement statistics.

Parameters	AtNRP2
**Data collection and Processing**	
Beam line	ID30A-3, ESRF, Grenoble, France
Detector type	Dectris pixel (Eiger_4M)
Wavelength (Å)	0.9794
Data Collection temperature (K)	100 K
Space Group	H32
α,β,γ a,b,c	90.00°, 90.00°, 120.00° 123.45 Å, 123.45 Å, 228.71 Å
Resolution (Å)	42.04–3.42 (3.50–3.42)
R_merge_ (%)	13% (51%)
I/σI	1.93
CC (1/2)	0.999 (0.722)
Total number of reflections	105,656 (10,947)
Completeness (%)	99.69 (100.00)
Multiplicity	11.4
Wilson B factor (Å^2^)	116.5
Average B, all atoms (Å2)	90.0
Anisotropy	0.027
Number of molecules in asymmetric unit	2
**Refinement**	
Number of unique reflections	9304 (914)
R _work_/R _free_ (%)	0.272/0.320 (0.277/0.327)
Total number of non-H atoms	2346
r.m.s. deviations: Bond lengths (Å) Bond angles (^o^)	0.004 0.96
Ramachandran plot values (%) Favored/Outliers	99.04/0.96

Values in parenthesis are for the highest resolution shell.

**Table 2:** Thermodynamic parameters as determined by ITC measurements.

Protein Complex	Kd1(nM)	Kd2(µM)	∆H1 (Kcal/mol)	∆H2 (Kcal/mol)	−T∆S1 (Kcal/mol)	−T∆S2 (Kcal/mol)	Reduced Chi Square Value
NRP1:H2A-H2B	130 ± 0.0005	2.4 ± 0.12	8.51 ± 0.107	6.00 ± 0.110	−17.9	−13.7	2.2 × 10^−2^
NRP2:H2A-H2B	39.6 ± 0.455	1.0 ± 0.0004	7.28 ± 0.04	1.33 ± 0.06	−17.4	−9.51	9.8 × 10^−3^

## Accession Numbers

Atomic coordinates and structure factor for the reported crystal structure has been deposited with the Protein Data Bank under accession number 6JQV.

## Supplementary Materials

The following are available online.
