# Supplementary material for: Structural Characterization of Arabidopsis thaliana NAP1-Related Protein 2 (AtNRP2) and Comparison with Its Homolog AtNRP1
Source: Molecules. 2019 Jun 17;24(12):2258. doi: 10.3390/molecules24122258 (PMC6630525; doi:10.3390/molecules24122258)
Supplement: Supplementary file 1 [file molecules-24-02258-s001.pdf]

Structural characterization of *Arabidopsis thaliana* NAP1-Related Protein 2 (AtNRP2) and comparison with its homolog AtNRP1

Ashish Kumar[1,2], Ajit Kumar Singh[1,2], Ruchir Chandrakant Bobde[1,3] and Dileep Vasudevan[1,*]

[1] Institute of Life Sciences, Bhubaneswar 751023, Odisha, India; mail2ashish.bt@gmail.com (A.K.); singh.ajit325@gmail.com (A.K.S.); rckngb30593@gmail.com (R.C.B.)
[2] Manipal Academy of Higher Education, Manipal 576104, Karnataka, India
[3] Regional Centre for Biotechnology, Faridabad 121001, Haryana, India
[*] Correspondence: dileep@ils.res.in. Tel.: +91-674-2304291; Fax: +91-674-2300728

Supplementary Table 1: List of all the species selected for sequence and phylogenetic analysis from both monocots and dicots

Organism	Protein Name	Gene Bank ID/NCBI Ref Seq/ Uniprot ID	Length (a.a)	Monocot/Dicot	Clade/Order
Actinidia chinensis	NRP	PSR89653.1	204	Dicot	Asterids, Ericales
Aegilops tauschii	NRP2	XP_020194932.1	251	Monocot	Commelinids, Poales
Amborella trichopoda	NRP2	XP_006827206.1	275	Dicot	Amborellales
Artemisia annua	NRP1	PWA88792.1	247	Dicot	Asterids
Ananas comosus	NRP2	OAY75887.1	256	Monocot	Poales
Arachis duranensis	NRP2	XP_015942681.1	265	Dicot	Rosids, Brassicales
Arachis ipensis	NRP2	XP_015942681.1	265	Dicot	Rosids
Arabidopsis lyrata	NRP1<br>NRP2	XP_020891472.1<br>XP_020870494.1	258<br>253	Dicot	Rosids
Asparagus officinalis	NRP2-like isoform X1	XP_020257311.1	253	Monocot	Asparagales
Arabidopsis	NRP1	NP_177596.1	256	Dicot	Brassicales

thaliana	NRP2	NP_564063.1	256		
Brachypodium distachyon	NRP2	XP_003575216.1	252	Monocot	Poales
Brassica napus	NRP2-like	XP_013657249.1	253	Dicot	Brassicales
Cajanus cajan	NRP2 isoform X1	XP_020234567.1	264	Dicot	Fabales
Carica papaya	NRP2-like	XP_021890590.1	263	Dicot	Brassicales
Capsella rubella	NRP1<br>NRP2 isoform X1	XP_006302716.1<br>XP_023645483.1	258<br>253	Dicot	Brassicales
Capsicum baccatum	NRP2	PHT47521.1	260	Dicot	Brassicales
Capsicum chinense	NRP2	PHU23992.1	260	Dicot	Solanales
Cephalotus follicularis	NAP domain containing protein	GAV57825.1	274	Dicot	Oxalidales
Chenopodium quinoa	NRP2 isoform X1	XP_021733819.1	260	Dicot	Caryophyllales
Citrus clementina	NRP2 isoform X1	XP_006439603.1	260	Dicot	Sapindales
Citrus sinensis	NRP2 isoform X1	XP_006476613.1	260	Dicot	Sapindales
Cucurbita maxima	NRP1-like<br>NRP2-like	XP_023001094.1<br>XP_022998832.1	264<br>268	Dicot	Cucurbitales
Cucurbita moschata	NRP1-like<br>NRP2-like	XP_022923755.1<br>XP_022949102.1	264<br>268	Dicot	Cucurbitales
Cynara cardunculus	NRP1 isoform X1<br>NRP2	XP_024972928.1<br>XP_024976694.1	264<br>247	Dicot	Asterales

Dichanthelium oligosanthes	NRP1<br><br>NRP2	OEL31860.1<br><br>OEL21843.1	255<br><br>190	Monocot	Poales
Durio zibethinus	NRP1-like<br><br>NRP2-like	XP_022768509.1<br><br>XP_022727878.1	262<br><br>261	Dicot	Malvales
Eutrema Salsugineum	NRP1<br><br>NRP2 isoform X1	XP_006390411.1<br><br>XP_006416566.1	260<br><br>260	Dicot	
Glycine soja	SET	KHN21849.1	261	Dicot	Fabales
Gossypium arboreum	SET	KHG17103.1	249	Dicot	Malvales
Helianthus annuus	NRP1-like<br><br>NRP2-like isoform X1	XP_021983899.1<br><br>XP_021978396.1	251<br><br>254	Dicot	Asterales
Hevea brasiliensis	NRP2-like	XP_021683068.1	266	Dicot	Malpighiales
Jatropha curcas	NRP2	XP_012080253.1	272	Dicot	Malpighiales
Lactuca sativa	NRP1<br><br>NRP2-like isoform X1	XP_023768179.1<br><br>XP_023743356.1	257<br><br>250	Dicot	Asterales
Manihot esculenta	NRP2<br><br>NRP2-like	XP_021616087.1<br><br>XP_021592793.1	263<br><br>265	Dicot	Malpighiales
Medicago truncatula	NRP1<br><br>NRP2-like Isoform X1	XP_013459455.1<br><br>XP_013457898.1	260<br><br>266	Dicot	Fabales
Momordica charantia	NRP1	XP_022137647.1	266	Dicot	Cucurbitales

Morus notabilis	NRP1	XP_010091501.2	264	Dicot	
Olea europaea var. sylvestris	NRP2-like	XP_022843635.1	261	Dicot	Lamiales
Oryza sativa japonica	NRP2	XP_015627496.1	262	Monocot	Poales
Oryza sativa indica	Nucleosome chromatin assembly protein	ABR26120.1	243	Monocot	Poales
Populus trichocarpa	NRP2<br><br>NRP2-isoform X1	XP_002323845.2<br><br>XP_024437616.1	261<br><br>272	Dicot	Malpighiales
Prunus avium	NRP1	XP_021820685.1	265	Dicot	Rosales
Prunus persica	NRP1	XP_007209477.1	265	Dicot	Rosales
Prunus yedoensis	NRP1-like	PQM41797.1	229	Dicot	Rosales
Quercus suber	NRP2-like isoform X1	XP_023927983.1	270	Dicot	Fagales
Ricinus communis	NRP2	XP_015582059.1	268	Dicot	Malpighiales
Rosa chinensis	NRP2-like	XP_024174323.1	265	Dicot	Rosales
Sesamum indicum	NRP2-like<br><br>NRP2-isoform X1	XP_011071637.1<br><br>XP_011087255.1	262<br><br>263	Dicot	Lamiales
Solanum tuberosum	NRP2-like	NP_001305500.1	268	Dicot	Solanales
Sorghum bicolor	NRP1<br><br>NRP2	XP_002447963.1<br><br>XP_002454059.1	257<br><br>251	Monocot	Poales
Spinacia oleracea	NRP2-like isoform X1	XP_021860196.1	262	Dicot	Caryophyllales
Theobroma cacao	NRP2	KHG17103.1	273	Dicot	Malvales

Trifolium pratense	SET-like	PNY13530.1	255	Dicot	Fabales
Vigna radiata	NRP2<br><br>NRP2-isoform X1	XP_014507147.1<br><br>XP_022635153.1	251<br><br>265	Dicot	Fabales
Zea mays	Uncharacterized	NP_001105066.1	251	Monocot	Poales
Ziziphus jujuba	NRP1<br><br>NRP2-like	XP_015883512.1<br><br>XP_015879370.1	263<br><br>255	Dicots	Rosales

Supplementary Table 2: Crystallization conditions, cryo-protectant conditions and diffraction limits for AtNRP2 crystals

Condition number	Crystallization condition composition	Cryo-protectant	Diffraction limit
AmSO4 Suite (3) [Fig 4a(i)]	0.2M Ammonium chloride	20% PEG 400	3.45 Å
AmSO4 Suite (25) [Fig 4a(ii)]	0.2 M tri-Potassium citrate, 2.2 M Ammonium sulfate	20% MPD	3.5Å
AmSO4 Suite (27) [Fig 4a(iii)]	0.2 M Potassium fluoride, 2.2 M Ammonium sulfate	20% glycerol	3.42Å
AmSO4 Suite (32) [Fig 4a(iv)]	0.2 M K/Na tartrate, 2.2 M Ammonium sulfate	20% ethylene glycol	No diffraction
AmSO4 Suite (40) [Fig 4a(v)]	0.2 M Sodium fluoride, 2.2 M Ammonium sulfate	20% MPD	No diffraction
AmSO4 Suite (91) [Fig 4a(vi)]	0.1 M Tris-sodium citrate, 2.4 M Ammonium sulfate	20% MPD	No diffraction

Supplementary Table 3: AtNRP2 NT/CT assembly status and stability as predicted by PDBePISA

Formula	Composition	Stable	Surface area ($Å^2$)	Buried surface area ($Å^2$)	ΔG(int)(Kcal/mol)	ΔG(diss)(Kcal/mol)

A_4	A_2B_2	Yes	33560	9100	-74.8	8.2
A_2	AB	Yes	18450	2800	-27.9	18.7

Supplementary Table 4: SAXS data collection and structure parameters

	AtNRP1 NT/CT	AtNRP2 NT/CT
**Data Collection Parameters**		
Beam line	BM29 BioSAXS (ESRF)	
Detector	PILATUS 1M	
Beam size (mm²)	0.77 X 0.77	
Wavelength (Å)	0.998	
q range ($Å^{-1}$)	0.4-49	
Sample Volume	50 µl	
Temperature	293 K	
Sample Concentration	2.0-5.0 mg/ml	
**Structural Parameters**		
I (0) { from Guinier}	48.9	39.84
R_g (Å) { from Guinier}	32.5	30.2
I (0) { from P (r)}	48.9	39.84
R_g (Å) { from P (r)}	32.5	30.2
D_{max} (Å)	97	97
Porod Volume estimate (V_p) ($Å^3$)	1.0383×10^5	8.537×10^4
**SAXS derived Molecular mass**		
From I (0) (kDa)	39.0	48.0
From Porod Volume (kDa)	64.8	53.3
From SAXSMow2 (kDa)	50.0	53.7
Molecular mass from sequence (kDa)	48.2	48.2

**Modeling Parameters**		
Symmetry	P1	P1
DAMAVER (20 DAMMIF P1) NSD*	0.914 ± 0.043	0.933 ± 0.071
**Software used**		
Primary data reduction	PRIMUSqt	
Data Processing	ATSAS	
Ab initio analysis	DAMMIF	
Validation and averaging	DAMAVER	
Computation of model intensity	CRYSOL	
Three dimensional graphic representation	PyMOL	

* Normalized Spatial Discrepancy

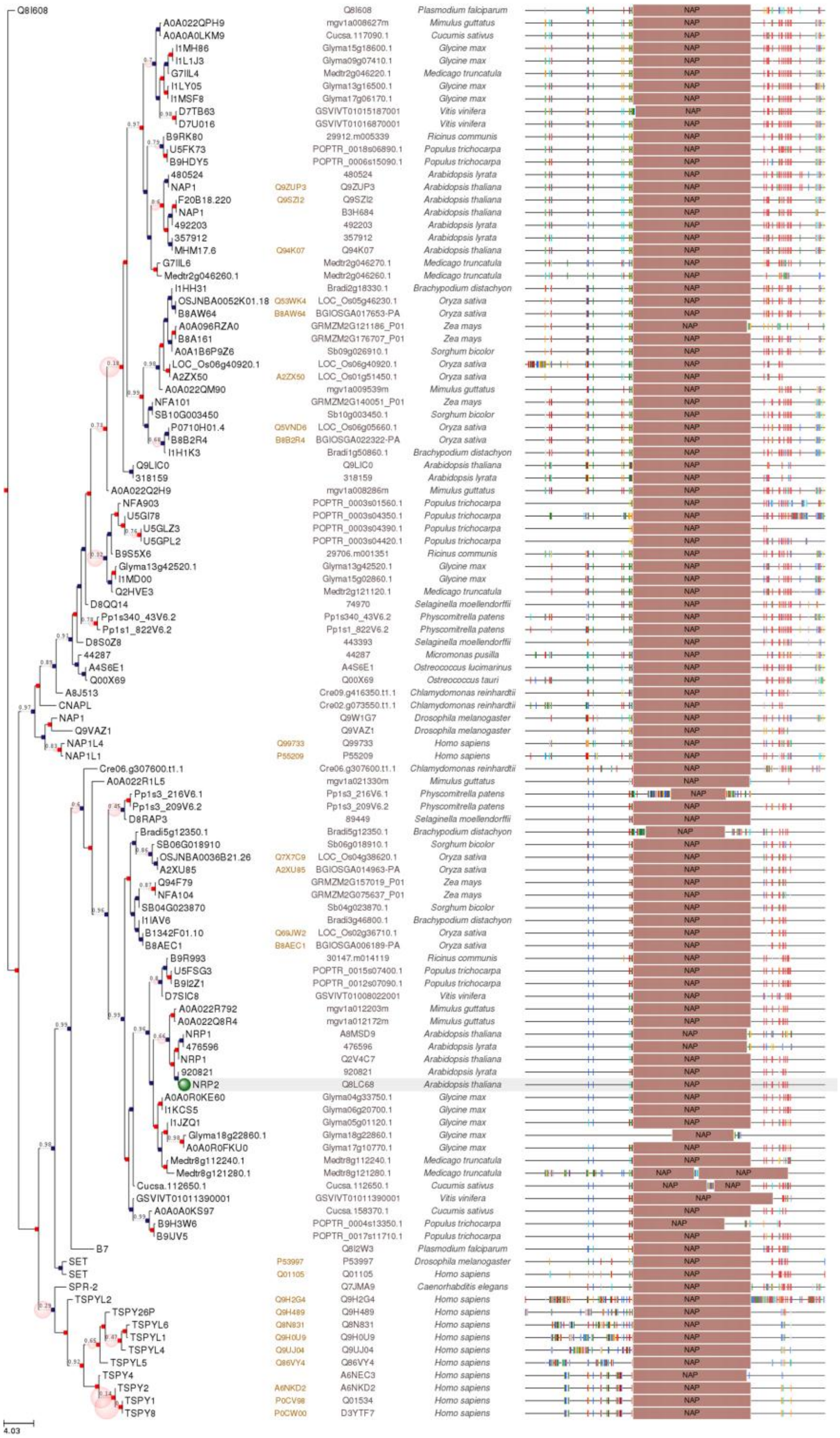

Supplementary Figure 1. Phylome of NAP-domain containing proteins. AtNRP2 sequence was used as the seed sequence to search all the similar domain containing proteins and their evolutionary relationship. The figure shows that the NAP and the NAP-related proteins (NRPs and SET domain

protein) have evolved separately because of gene duplication in a single ancestral gene, followed by independent speciation events.

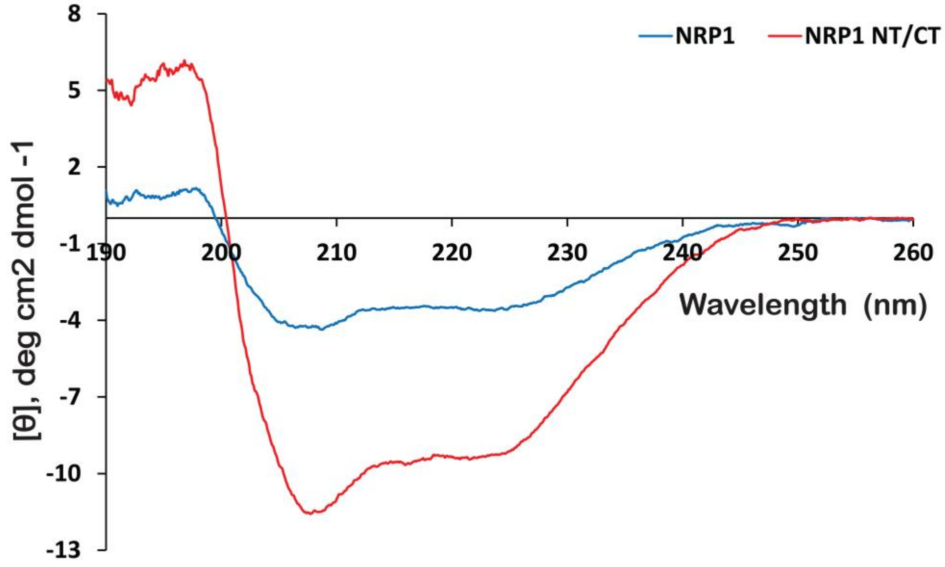

Supplementary Figure 2. The near-UV Circular dichroism (CD) spectroscopy profile of full-length AtNRP1 and AtNRP1 NT/CT, showing those to be predominantly α-helical in composition. The CD spectrum was measured using a J-1500 Circular Dichroism spectrophotometer (Jasco).

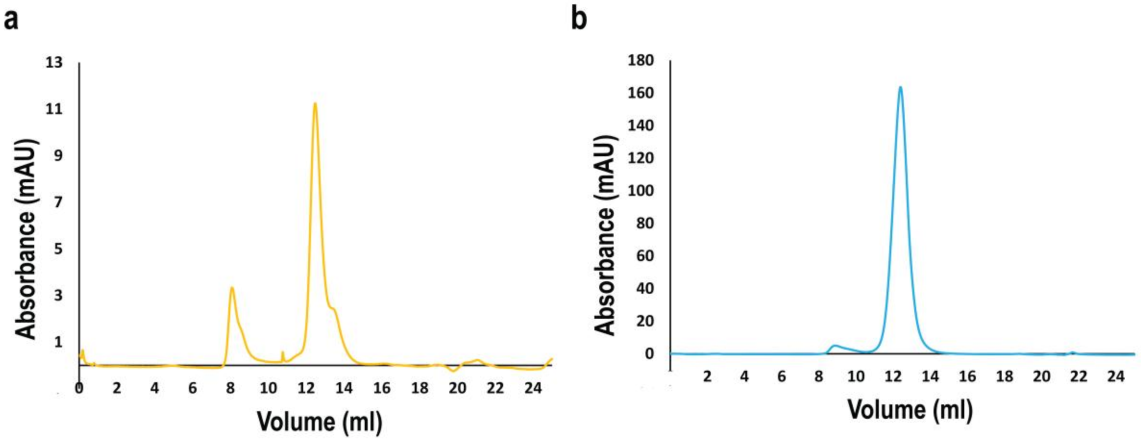

Supplementary Figure 3. Thermal stability of full-length AtNRP1 and full-length AtNRP2 at 60°C. **a** Chromatogram for AtNRP1 after heating at 60°C for 10 minutes**. b** Chromatogram for AtNRP2 after heating at 60°C for 10 minutes**.** AtNRP1 shows some amount of aggregated protein in void volume, however, AtNRP2 showed only milder aggregation.

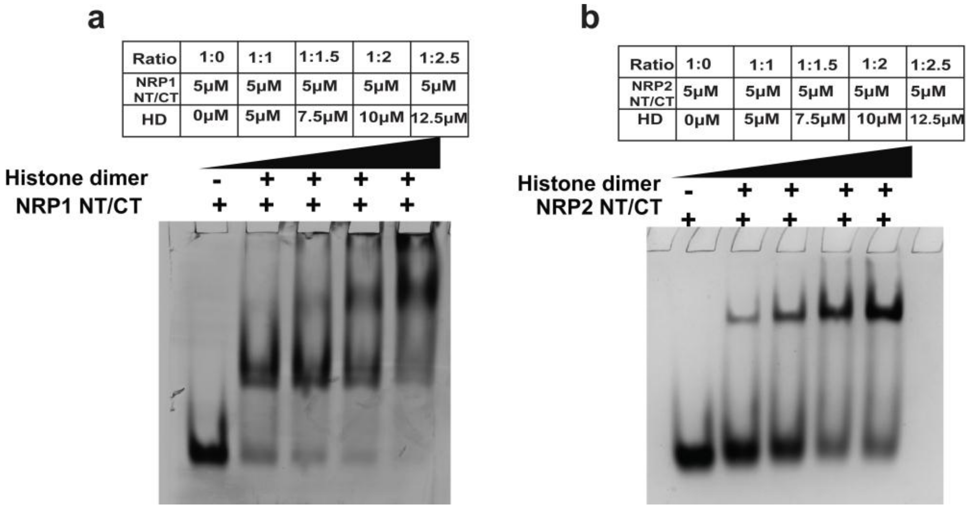

Supplementary Figure 4. Interaction of AtNRP1 NT/CT and AtNRP2 NT/CT with histone H2A-H2B dimer by electrophoretic mobility shift assay. **a** AtNRP1 NT/CT**. b** AtNRP2 NT/CT**.** H2A-H2B dimer is given as Histone dimer (HD) in the figure.
